# New names and status for Pacific spiny species of *Solanum* (Solanaceae, subgenus Leptostemonum Bitter; the Leptostemonum Clade)

**DOI:** 10.3897/phytokeys.145.48531

**Published:** 2020-04-10

**Authors:** Donald H.R. McClelland, Michael Nee, Sandra Knapp

**Affiliations:** 1 Environmental Science, Bard College at Simon’s Rock, 84 Alford Road, Great Barrington, MA 01230, USA Bard College at Simon’s Rock Great Barrington United States of America; 2 Missouri Botanical Garden, P.O. Box 299, St. Louis, MO 63166, USA Missouri Botanical Garden St. Louis United States of America; 3 Department of Life Sciences, Natural History Museum, Cromwell Road, London SW7 5BD, UK Natural History Museum London United Kingdom

**Keywords:** Endemism, Fiji, Hawaiian islands, New Caledonia, New Guinea, new species, Pacific islands, Solomon Islands, Vanuatu

## Abstract

Five new species of spiny solanums (Solanum subgenus Leptostemonum Bitter; the Leptostemonum Clade) are described from the islands of the Pacific. Two of the new species are from Fiji (*S. pseudopedunculatum* D.McClelland, **sp. nov**. and *S. ratale* D.McClelland, **sp. nov**.), two from New Caledonia (*S. memoayanum* D.McClelland, **sp. nov.** and *S. semisucculentum* D.McClelland, **sp. nov**.), one from Papua New Guinea (*S. labyrinthinum* D.McClelland, **sp. nov**.) and another from Vanuatu (*S. vanuatuense* D.McClelland, **sp. nov**.). A new status and combination is provided for the rare Hawaiian endemic *S. caumii* (F.Br.) D.McClelland, **comb. et stat. nov**. and a new type designated for *S. peekelii* Bitter of Papua New Guinea, for which a description is also provided. All species are illustrated with digitized herbarium specimens, mapped and have been assigned a preliminary conservation status using current IUCN guidelines. Details of all specimens examined are provided in a Suppl. materials 1: file SM1.

## Introduction

*Solanum* L. (Solanaceae) is one of the largest genera of angiosperms (Frodin 2004), with ca. 1,400 species occurring on all continents except Antarctica. Species of *Solanum* occur in a wide variety of habitats from deserts to tropical rainforests, and the highest species-level diversity occurs in South America. The last global monographic treatment of *Solanum* dates from the 19^th^ century (Dunal 1852), which included 901 species (with an additional 19 incompletely known). *Solanum* taxonomy has proceeded in a piecemeal fashion until relatively recently and the genus had acquired a reputation of being intractable, but recent monographic work has begun to remedy this situation (e.g., Vorontsova and Knapp 2016).

The largest monophyletic group of *Solanum*, known as the Leptostemonum clade or S. subgenus Leptostemonum Bitter (Bohs 2005; Weese and Bohs 2007; Stern et al. 2011), includes prickly plants with stellate indumentum (the “spiny” solanums) and comprises approximately half the species diversity of the genus. It consists of a large lineage of approximately 570 species, of which approximately half are primarily New World in distribution, with significant diversity found in the Old World (including Oceania). Approximately 240 species are confined to the Old World tropics (see Aubriot et al. 2016), and a significant center of spiny solanum diversity is found in Australia (Symon 1981). The scattered islands of the Pacific Ocean are not a center of diversity for the spiny solanums, but the taxa found there are often highly endemic (see species described herein). The spiny solanums occurring in the Pacific were considered to belong to the traditional sections *Dunaliana* Bitter and *Irenosolanum* Bitter (Bitter 1917, 1922), and were grouped in the *S. dunalianum* and *S. sandwichense* species groups by Whalen (1984). Using a combination of morphological and molecular characters the species of these groups have been shown to fall into two monophyletic clades corresponding to Bitter’s sections (McClelland 2012), but their members have generally not been included in wider molecular analyses of Old World spiny solanums (e.g., Aubriot et al. 2016). Those few that have been included (i.e., *S. sandwichense* Hook., *S. incompletum* Dunal, and *S. pancheri* Guillamin of section Irenosolanum sensu McClelland 2012; *S. dunalianum* Gaudich. of section Dunaliana sensu McClelland 2012) are nested within the larger “Sahul-Pacific clade” of Aubriot et al. (2016) that is itself sister to the rest of the Old World spiny solanums (sensu Stern et al. 2011). They are not recovered as either monophyletic or as sister to each other. Aubriot et al. (2016) resolve *S. dunalianum* as a member of a group with *S. lianoides* Merr. & L.M.Perry and *S. graciliflorum* Dunal of the Philippines and Indonesia respectively, neither of which was considered by McClelland (2012), while the other three taxa are sequential sisters to a clade of New Guinea species (see fig. 3 of Aubriot et al. 2016). Relationships of these Pacific taxa are not clear yet, and their inclusion in wider phylogenetic analyses with broad sampling of Australasian taxa is a priority.

Despite previous floristic work in the Pacific (e.g., Seemann 1866; Bitter 1917, 1921; Symon 1985, 1999; Smith 1991) the spiny solanums of the Pacific are relatively poorly known compared to other areas. Here we describe five taxa recognized as new during the course of monographic work (McClelland 2012; D.H.R. McClelland and M. Nee, in prep.), recognize a variety of a Hawaiian endemic at the species level, and provide a new type and description for a misunderstood species from Papua New Guinea. We also provide preliminary conservation assessments for each of these species.

## Materials and methods

The species descriptions and circumscriptions here are based on examination of herbarium specimens from 24 herbaria (103 collections, 266 specimens) worldwide (A, BH, BISH, BM, CANB, G, GH, K, L, LAE, MEL, MO, NOU, NSW, NY, P, PTBG, RSA, S, SING, U, UC, US, W) and field work undertaken in Hawaii and New Caledonia by DHRM and MN in 2009. All specimen data are presented in the Suppl. materials 1: file SM1 and on the Natural History Museum Data Portal (https://doi.org/10.5519/0072409).

To assess the conservation status of the species treated here Extent of Occurrence (EOO) and Area of Occupancy (AOO) were calculated using GeoCAT (www.geocat.kew.org) with a 2 km cell width for AOO calculation. The preliminary conservation status was assessed using the IUCN (2019) criteria based on the GeoCAT analyses (Moat 2007; Bachman et al. 2011) combined with field knowledge. All specimens examined are cited in the text. Our delimitation of these new taxa is based on the “morphological species concept” (Davis and Heywood 1963; Mallet 1995).

## Taxonomic treatment

### Section Dunaliana Bitter (sensu McClelland 2012)

#### Solanum labyrinthinum

Taxon classificationPlantaeSolanalesSolanaceae

D.McClelland sp. nov.

EC439C69-64C6-59E1-9A58-4C1ED0296AA4

urn:lsid:ipni.org:names:77209325-1

Figure 1

##### Diagnosis.

Like *S. dunalianum* Gaudich. but with narrowly elliptic to lanceolate leaves, many-branched inflorescences arising from the middle of the internode, and smaller fruits.

##### Type.

Papua New Guinea. Milne Bay Province: Normanby Island, Waikaiuna [Bay], 5 m, 17 Apr 1956 (fl, fr), *L.J. Brass 25460* (holotype: LAE [acc. # 47392]; isotypes: A, K [K000922591], L [L.4307630], S, US [acc. # 2408171]).

**Figure 1.**
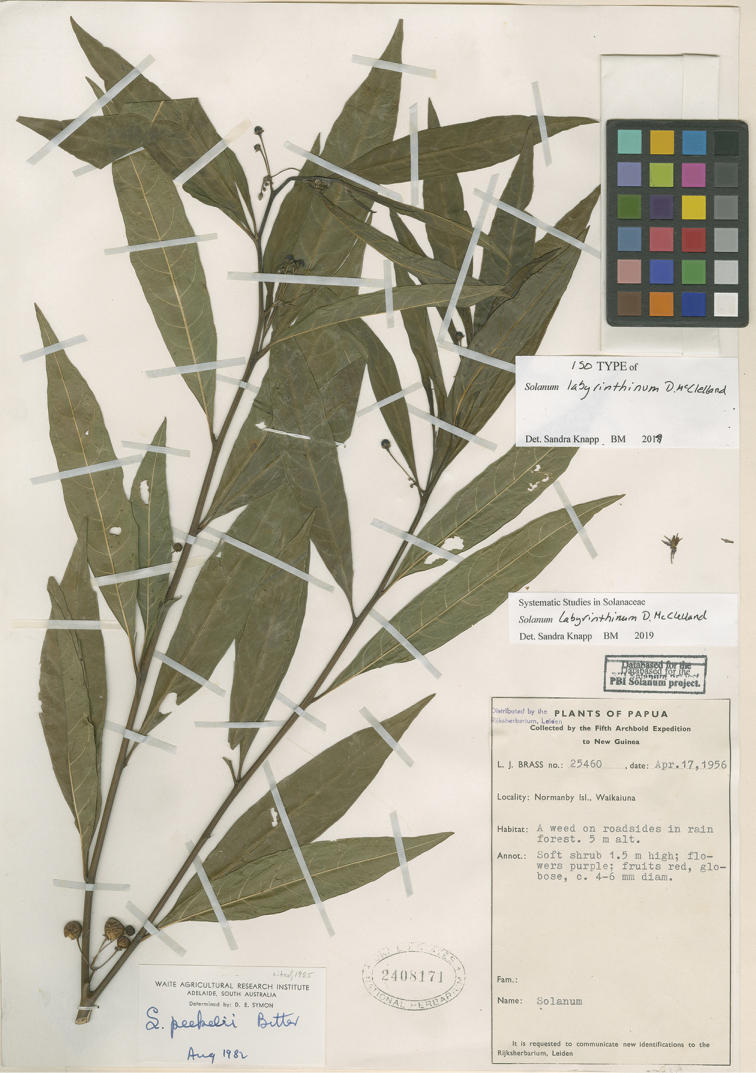
*Solanum labyrinthinum* D.McClelland (isotype *Brass 25460*, US [acc. # 2408171]). Reproduced with permission.

##### Description.

Erect shrub to ca. 2.5 m, the internodes to 6.5 cm long, apparently unarmed. Stems with a few scattered ferruginous sessile porrect-stellate trichomes when young, the rays 6–7, ca. 0.15 mm long, the midpoint more or less equal to the rays, the stems soon glabrescent; new growth sparsely pubescent with sessile stellate trichomes and minute glandular papillae; bark of older stems pale gray. Sympodial units difoliate, the leaves geminate or not, if geminate members of a pair more or less equal in size and shape. Leaves simple; blades 8.0–16.0 cm long, 1.4–3.7 cm wide, ca. 4.5–6.0 times as long as wide, narrowly elliptic to lanceolate, subcoriaceous, concolorous, unarmed; adaxial surfaces glabrous or with a few sessile porrect-stellate trichomes with 5–8 rays 0.15–0.2 mm long, the midpoints much shorter than the rays; abaxial surfaces glabrous; principal veins 7–10 pairs, the midrib raised both abaxially and adaxially, the lateral veins weakly brochidodromous, raised both abaxially and adaxially, drying yellowish or dark; base cuneate; margins entire or slightly wavy; apex acute to somewhat acuminate; petiole 0.9–1.8 cm long, 0.8–1.3 mm in diameter, channeled adaxially, glabrous when mature, unarmed. Inflorescence to 7.5 cm long, appearing lateral, extra-axillary, unbranched to forked to many-branched, with few to ca. 50 flowers, sparsely to moderately pubescent with sessile porrect-stellate trichomes when young, soon glabrous, unarmed; peduncle 0.1–0.6 cm long,, unarmed; pedicels 0.6–1.0 cm long, ca. 0.3 mm in diameter at the base, ca. 0.5 mm in diameter below the calyx, straight, gradually increasing in diameter from the base distally, articulated at the base; pedicel scars congested to spaced 0.9 mm apart, rigid, in two rows. Buds conical, the calyx enclosing the corolla when young, the corolla sparsely pubescent with scattered stellate trichomes and glandular papillae on exposed abaxial surfaces, strongly exserted from the calyx lobes before anthesis. Flowers 5-merous, all perfect. Calyx 1.4–1.7 mm long, with the tube to 0.8–1.2 mm long, in bud appearing nearly truncate with apiculate lobe tips to 0.5 mm long, splitting in the scarious sinuses at anthesis, the lobes 1.1–1.7 mm long, 1–1.4 mm wide, deltate, abaxially glabrous to sparsely pubescent with scattered porrect-stellate trichomes and minute glandular papillae, adaxially glabrous. Corolla ca. 1.2 cm in diameter, purple or deep violet, stellate, the interpetalar tissue not well-developed, the lobes 3.7–5.4 mm long, 1.6–2 mm wide, long-deltate, spreading at anthesis. abaxially densely stellate pubescent where exposed in bud, adaxially glabrous or with a few stellate trichomes near the tips. Stamens equal; filament tube minute; free portion of the filaments ca. 0.5 mm long, glabrous; anthers 3.5–4 mm long, 0.6–1 mm wide, tapering, connivent, yellow, poricidal at the tips, the pores directed distally. Ovary conical, glabrous; style 3.0–5.0 mm long, ca. 0.2 mm in diameter, exserted from or equal to the anther cone, filiform, straight, glabrous; stigma ca. 0.4 mm in diameter, bilobed, minutely papillate. Fruit a globose berry, 4.0–6.0 mm in diameter, red when mature, glabrous, the pericarp thin, shiny, opaque; fruiting pedicels 1.2–1.6 cm long, 0.4–1 mm in diameter at the base, 0.8–1.1 mm in diameter at the apex, erect; fruiting calyx not accrescent, the lobes 1.4–2.2 mm long, 1.0–1.5 mm wide, glabrous, appressed to the berry surface or slightly reflexed. Seeds 10–40 per berry, 1.8–2.5 mm long, 1.5–1.8 mm wide, flattened, suborbicular to reniform, notched at the point of attachment, yellow-ferruginous when dry, the surface minutely pitted (alveolate), the testal cells with straight walls. Chromosome number not known.

##### Distribution and ecology

**Figure 2.**
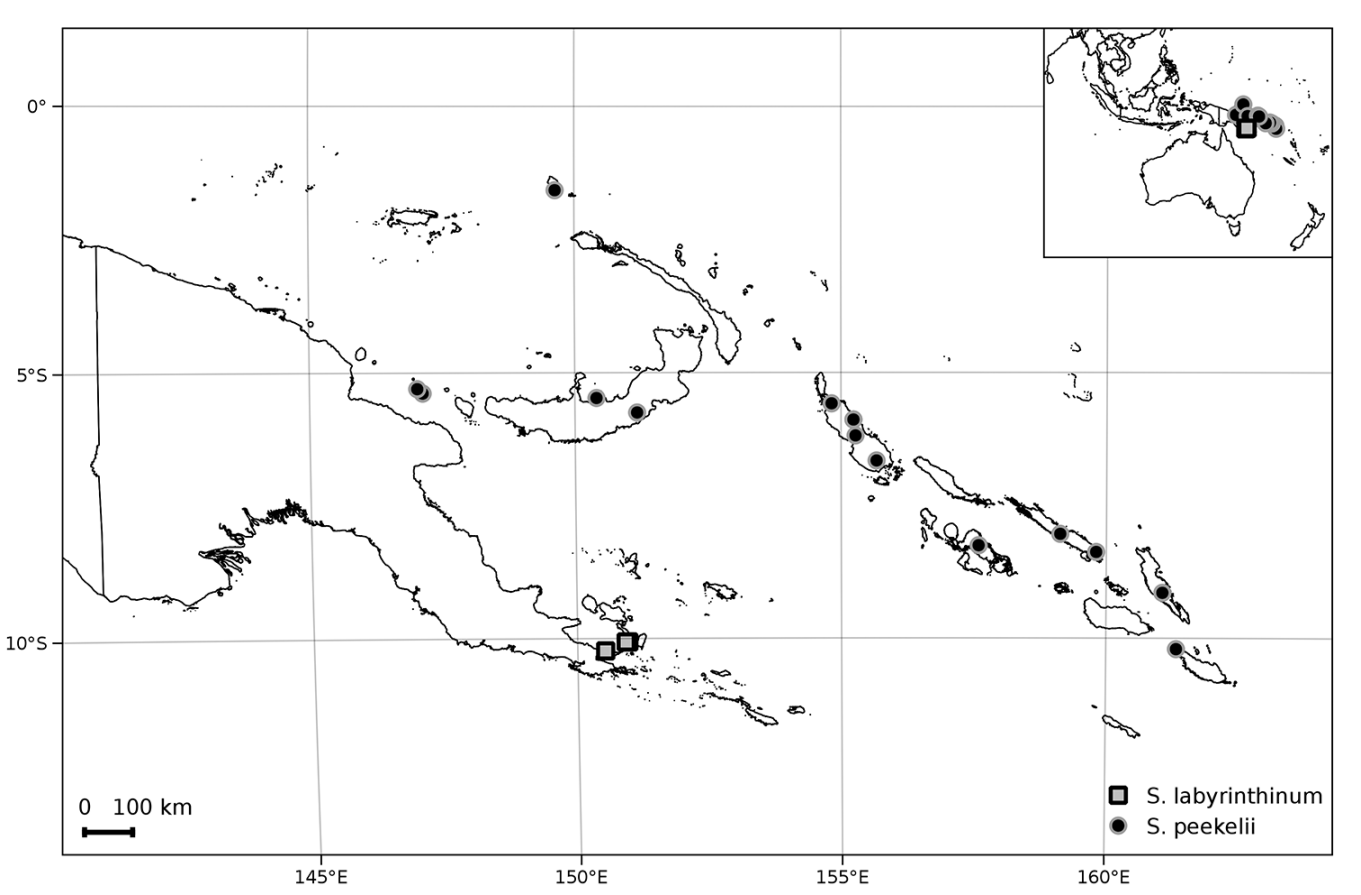
Distribution of *Solanum labyrinthinum* and *S. peekelii*.

(Figure 2). *Solanum labyrinthinum* is known from Normanby Island and adjacent East Cape on New Guinea; it has been collected along roads in rainforest from 5 to 185 m elevation.

##### Phenology.

Known to flower and fruit in Apr. Perhaps fertile year round like the other members of Solanum section Dunaliana.

##### Etymology.

The specific epithet was chosen to reflect the taxonomic labyrinth involving two other species of New Guinea *Solanum* that was unintentionally created by Symon (1985).

##### Preliminary conservation assessment

(IUCN 2019). EOO = 51 km^2^ [CR – Critically Endangered]; AOO = 12 km^2^ [EN – Endangered]. We assess *Solanum labyrinthinum* as EN (Endangered) using IUCN Criteria B1a,b, due to its restricted distribution, its primary forest habitat and lack of recent collections indicating population decline. Although it is found both on New Guinea and in the Solomon Islands, the destruction of lowland forest habitat in coastal areas suggests conservation concern.

##### Discussion.

*Solanum labyrinthinum* differs from other members of section Dunaliana (sensu McClelland 2012) in its almost completely glabrous narrowly elliptic or somewhat lanceolate leaves. Like *Solanum torricellense* Bitter of New Guinea, but unlike the other members of this group (section Dunaliana sensu McClelland 2012), the inflorescence of this species emerges from the middle of the internode. *Solanum labyrinthinum* differs from *S. peekelii* with which it has been confused (see below) in its narrow completely glabrous leaves at maturity with distinctive appressed stellate trichomes when young, its fewer principal leaf veins (7–10 versus 10–14) that often dry yellowish, its slightly smaller flowers (ca. 1.2 cm in diameter versus to 1.5 cm in diameter) and fruits, and in its distribution. The stems of *S. labyrinthinum* are often purplish tinged, and the flowers are darker purple than those of *S. peekelii*.

Symon (1985) applied the name *S. peekelii* to the plants we here recognise as *S. labyrinthinum*, then applied the name *S. torricellense* to plants matching the description of *S. peekelii* and provided a new name for plants matching the type of *S. torricellense*, thus creating a taxonomic labyrinth (see below under typification of *S. peekelii* for details, and Solanaceae Source (www.solanaceaesource.org) for descriptions of the other taxa involved).

##### Additional specimens examined.

Papua New Guinea. Milne Bay Province: Awaiama, Sep 1895 (fr), *Fitzgerald 16* (MEL); Normanby Island, Sewa Bay, 600 ft, 20 Apr 1956 (fl), *J. Womersley & L. Brass NGF-8678* (A [2 sheets], BM [2 sheets], BO, BRI, CANB, K, L, LAE, NSW, SING).

#### Solanum peekelii

Taxon classificationPlantaeSolanalesSolanaceae

Bitter, Bot. Jahrb. Syst. 55: 73. 1917

9205D7E7-A007-5C78-A739-0776E167A276

Figure 3

Solanum dunalianum var. inerme Witasek, Denkschr. Akad. Wiss. Wien 89: 601. 1914. Type: Papua New Guinea. Bougainville (North Solomons): “Salomonsinseln, Insel Bougainville, Am Strande bei dem Eingebornen dorf Numa-Numa”, Sep 1905, *K. Rechinger & L. Rechinger 3607* (lectotype, designated here: W [acc. # 1916-10316]; isolectotype: W [acc. # 1916-10317]).

##### Type.

Papua New Guinea. New Ireland Province: Bismark Archipelago, New Ireland [“Neu-Mecklenburg”], Buragamata near Namatanai, 12 m, Jul, *G. Peekel 523* (holotype: B [destroyed]). Papua New Guinea. New Britain Province: West New Britain, West Nakanai, Malalia near Cape Hoskins, 16 Aug 1954 (fl, fr), *A. Floyd NGF-6549* (neotype, designated here: LAE [acc. # 16333]; isoneotypes: A, BM [BM000886258], CANB [acc. # 75841.1], K [K000922697], L [L.4307653], MEL [MEL0625408A], NSW [acc. #594233], US [acc. # 2211029, barcode 02838857).

**Figure 3.**
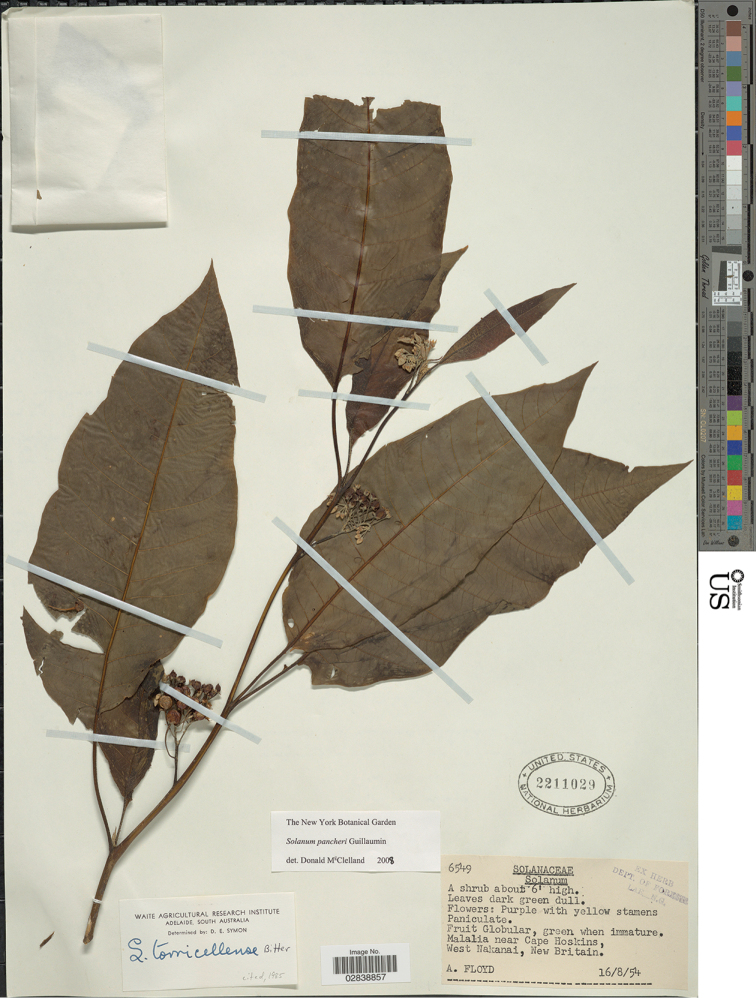
*Solanum peekelii* Bitter (isoneotype *Floyd NGF-6595*, incorrectly labelled as *S. pancheri*, US [acc. # 2211029, barcode 02838857]). Reproduced with permission.

##### Description.

Erect shrub or small tree to 4(5) m tall, the internodes to 13.5 long, unarmed to sparsely armed. Stems pubescent with yellow, sessile porrect-stellate trichomes, the rays 6–10, 0.15–0.2 mm long, the midpoint shorter than to more or less equal to the rays; new growth pubescent near the leaf bases with sessile porrect-stellate trichomes like those of the stems, soon glabrescent, the prickles if present 1–2 mm long, weak-walled, somewhat broadened at the base, very sparse; bark reddish brown, glabrescent, somewhat shiny, unarmed or with very sparse prickles 1–2 mm long. Sympodial units difoliate, the leaves geminate, members of a pair more or less equal in size and shape. Leaves simple; blades 10.0–25.5 cm long, 5.0–9.0 cm wide, 2.4–3.2 times as long as wide, obovate to elliptic, chartaceous, concolorous, unarmed; adaxial surfaces more or less glabrous, the pubescence denser near the basal portion of the blade, the trichomes porrect-stellate, the rays (5)6–8, 0.5–0.2 mm long, the midpoint shorter than or equal to the rays; abaxial surfaces sparsely pubescent with sessile porrect-stellate trichomes with 6–8-rays 0.2–0.3 mm long, the midpoint erect, shorter than to more or less equal to the rays; principal veins 10–14 pairs, the midrib raised abaxially and adaxially, the lateral veins weakly brochidodromous, raised abaxially and adaxially; base rounded to cuneate, occasionally long attenuate in vigorous growth, sometimes somewhat oblique; margins entire or slightly wavy; apex acuminate; petiole 1.6–5.0 cm long, 1.0–2.2 mm in diameter, channeled above, moderately stellate-pubescent when young, unarmed, the pubescence becoming sparse to moderate and restricted to the adaxial channel with age. Inflorescence to 1.1–6 cm long, appearing lateral, extra-axillary, in the upper 1/3 of the internode often just below the leaf, unbranched or forked with a single pedicel attached below the fork, with up to 45 flowers, densely pubescent with sessile porrect-stellate trichomes like those of the stems; peduncle 0.7–1.2 cm long; pedicels 0.4–1.2 cm long, 0.3–0.4 mm in diameter at the base, 0.5–0.7 mm in diameter below the calyx, straight, gradually increasing in diameter from the base distally, articulated at the base; pedicel scars congested to spaced ca. 0.6 mm apart, rigid, in two rows. Buds conical, the calyx moderately to densely stellate-pubescent, the corolla densely stellate-pubescent where exposed abaxially, only somewhat exserted from the calyx before anthesis. Flowers 4–5-merous, all perfect or apparently so. Calyx 1.6–2.3 mm long, the tube 1.3–1.8 mm long, in bud appearing nearly truncate with lobe tips apiculate or reduced to minute protuberances 0.1–1.1 mm long, the sinuses transparent when dry, the lobes 0.7–1.7 mm long, 0.9–1.5 mm wide at anthesis, deltate, splitting in the sinuses at anthesis, moderately to densely pubescent abaxially with sessile porrect-stellate trichomes, glabrous adaxially, Corolla 1–1.5 cm in diameter, stellate, white or lavender to purple, the interpetalar tissue poorly-developed, glabrous, the lobes 5.7–6.5 mm long, 2.8–3.1 mm wide, triangular, apparently spreading at anthesis, densely pubescent along the petal midvein abaxially, more or less glabrous adaxially. Stamens equal; filament tube minute, with tiny teeth between each filament (fide Bitter 1917); free portion of the filaments ca. 0.6 mm long, glabrous; anthers 2.2–4.8 mm long, 0.6–0.9 mm wide, tapering, yellow, connivent, poricidal at the tips, the pores dorsally directed. Ovary 0.4–0.6 mm in diameter, ovoid, glabrous or with a few sessile porrect-stellate hairs at the very apex; style 3.7–5.4 mm long, 0.2–0.3 mm in diameter, filiform, straight, sparsely to moderately stellate pubescent at the very base to basal 1/3; stigma ca. 0.4 mm in diameter, capitate or slightly bilobed. Fruit a globose juicy berry, 4.0–7.0 mm in diameter, the immature fruits green, maturing red, glabrous, the pericarp thin, somewhat shiny, opaque; fruiting calyx lobes 1.2–2.3 mm long, 1.4–2.0 mm wide, sparsely to moderately pubescent, appressed in ripe fruit; fruiting pedicels 9.2–15.2 mm long, 0.3–0.7 mm in diameter at the base, 0.7–1.3 mm in diameter below the calyx, straight, gradually increasing in diameter from the base distally;. Seeds ca. 45 per fruit, 1.6–2.3 mm long, 1.4–1.9 mm wide, flattened, orbicular to reniform, notched at the point of attachment, yellow-ferruginous when dry, the surface the central area nearly smooth, the margins minutely pitted, the testal cells pentagonal or rectangular. Chromosome number not known.

##### Distribution and ecology

(Figure 2). *Solanum peekelii* occurs from the Bismarck Archipelago of Papua New Guinea to the Solomon Islands. It has been collected in disturbed habitats such as old village gardens, forest edges, and secondary forest, from sea level to 170(–1070) m elevation. According to Peekel (1984), *S. peekelii* is one of the first plants to appear in abandoned gardens; the lectotype of S. dunalianum var. inerme was collected on the beach near a village.

##### Common names and uses.

Papua New Guinea. Bougainville: Kala-bu-ku (*Kajewski 1790*); Madang: Airoroana (*Lepofsky 466*), New Georgia: Susuriata (*Waterhouse 31*), leaves said to be used in preparation of a cough medicine. Peekel (1984) reported the Pala people used stems from the species to construct graters for taro and yams by binding them crosswise to a grid.

##### Preliminary conservation assessment

(IUCN 2019). EOO = 477,504 km^2^ [LC – Least Concern]; AOO = 52 km^2^ [EN – Endangered]. We assess *Solanum peekelii* as LC (Least Concern) due to its relatively wide distribution (EOO) and its occurrence in secondary and other disturbed habitats. Its use by people may also afford it some protection.

##### Discussion.

*Solanum peekelii* was named for P. Gerhard Peekel (1876–1949), a missionary in New Guinea who spent 43 years collecting plants in the Bismarck Archipelago, primarily on New Ireland, resulting in a flora of the region (Peekel 1984). Though Peekel did not consider himself a botanist, he was clearly dedicated to the plant diversity of the area. At the outbreak of the Second World War he took measures to preserve his manuscript. Notwithstanding, it suffered from the unfavorable conditions in dugouts and concentration camps; one volume was even pierced by a bullet from an airplane. During the Japanese occupation of New Ireland, Peekel’s botanical knowledge saved his life. All people of European descent were brought to a concentration camp where Peekel was the only male not to be killed because the Japanese relied on his botanical knowledge (Peekel 1984).

Symon (1985) mistakenly treated *S. peekelii* under the name *S. torricellense*, from which it differs in its smaller berries (4–7 mm versus 7–9 mm in diameter) and sparser pubescence of stellate rather than the multangulate trichomes characteristic of *S. torricellense*. Symon (1985) applied the name *S. peekelii* to plants of what we have named here *S. labyrinthinum*. He then provided a new name, *S. mankiense* Symon, for plants matching the type of *S. torricellense* (see discussion under those species on Solanaceae Source, www.solanaceaesource.org). Because the type of *S. peekelii* was presumed destroyed at B, Symon (1985) designated *Womersley & Brass NGF-8678* from Normanby Island (here included in *S. labyrinthinum*) as the neotype of *S. peekelii*, adding a final twist to this taxonomic labyrinth. However, none of the duplicates of *Womersley & Brass NGF-8678* agree with Bitter’s (1917) detailed protologue of *S. peekelii* in the pubescence of the leaves and stem, leaf size or length to width ratio, number of lateral leaf vein pairs, corolla size, and lack of small teeth between each filament. Additionally, the travels and collecting habits of P.G. Peekel are well-documented (Peekel 1984), and he never journeyed to Normanby Island. The protologue of *S. peekelii* and the neotype designated by Symon (1985) are thus serious conflict, and the neotypification of *S. peekelii* with *Womersley & Brass NGF-8678* should be superseded (Turland et al. 2018, Art. 9.19c); we neotypify the species again here with a specimen from New Ireland that exactly matches Bitter’s protologue and comes from an area where Peekel is documented to have collected.

##### Specimens examined.

Papua New Guinea. Bougainville: Kugu-maru, Buin, 150 m, 1 Jun 1930 (fl, fr), *Kajewski 1790* (A, BM, BRI, G, P); forest edge at the village Numa-Numa, Sep 1905 (fl, fr), *Rechinger & Rechinger 5365* (W); vic. of Barilo village, ca. 6 miles N of Buin Station, ca. 500 ft, 28 Aug 1964 (fl, fr), *Schodde & Craven 3938* (A, CANB, K, L, LAE); Namatoa, ca. 1500 ft, 7 Mar 1932 (fl), *Waterhouse 691* (K). East New Britain: Sub-district Gasmata, Torlu River, ca. 3500 ft, 27 Mar 1965 (fl, fr), *Sayers NGF-24265* (A, K, L, LAE). Madang: Sub-district Saidor, Long Island, beach of Lake Wisdom, 400 m, 4 Oct 1971 (fl, fr), *Essig & Lelean LAE-55039* (A, BH, CANB, K, L, LAE, NY); Sub-district Saidor, Long Island, 5 miles N of Matafuma Village, 50 ft, 15 Nov 1969 (fr), *Vandenberg & Katik NGF-42331* (A, CANB, K, L, LAE). New Georgia: sin. loc., 21 May 1929 (fr), *Waterhouse 31* (K, LAE). New Ireland: St. Matthias Group, Eloaua, 30 m, 16 Nov 1968 (fr) *Lepofsky 466* (BISH).

Solomon Islands. Makira. Maru Bay, 550 ft, 28 Nov 1968 (fr), *Gafui 12869* (K, L, LAE). Malaita: NE Malaita, 450 ft, 21 Nov 1968 (fl, fr), *Mauriasi 13463* (K, L); Su’u area, SE Malaita, 500 ft, 2 Dec 1968 (fl, fr), *Mauriasi 13601* (A, K, L, LAE). Santa Isabel: Suwa, Toabul, 26 Nov 1932 (fl, fr), *Brass 3231* (A, L); head of Tatamba Bay, sea level, 1 Jan 1965 (fl, fr), *Hunt 2835* (A, K, L, LAE, P).

### Section Irenosolanum Bitter (sensu McClelland 2012)

#### Solanum caumii

Taxon classificationPlantaeSolanalesSolanaceae

(F.Br.) D.McClelland, comb. et stat. nov.

F19C4610-8F68-5AEF-99A4-7758D76A20F2

urn:lsid:ipni.org:names:77209326-1

Figure 4

Solanum nelsonii Dunal var. caumii F.Br., Bull. Bish. Mus. 81: 36, pl. 15B. 1931. Type. United States of America. Hawaii: Leeward Islands, Nihoa, 75 m, 19 Jun 1923 (fl, fr), *E. Caum 84* (holotype: BISH [acc. # 581193, barcode BISH1014622]; isotypes: BISH [acc. # 581194, barcode BISH1014623], K [K000922665], NY [00172288], UC [acc. #505079]).
Solanum nelsonii Dunal var. acuminatum F.Br., Bull. Bish. Mus. 81: 36, pl. 16A. 1931. Type. United States of America. Hawaii: Leeward Islands, Nihoa, 75 m, 19 Jun 1923 (fl, fr), *E. Caum 68* (holotype: BISH [acc. # 581186, barcode BISH1014620]; isotypes: BISH [acc. # 581185, barcode BISH1014621], K [K000922664], NY [00172287]).

##### Type.

Based on Solanum nelsonii Dunal var. caumii F.Br.

**Figure 4.**
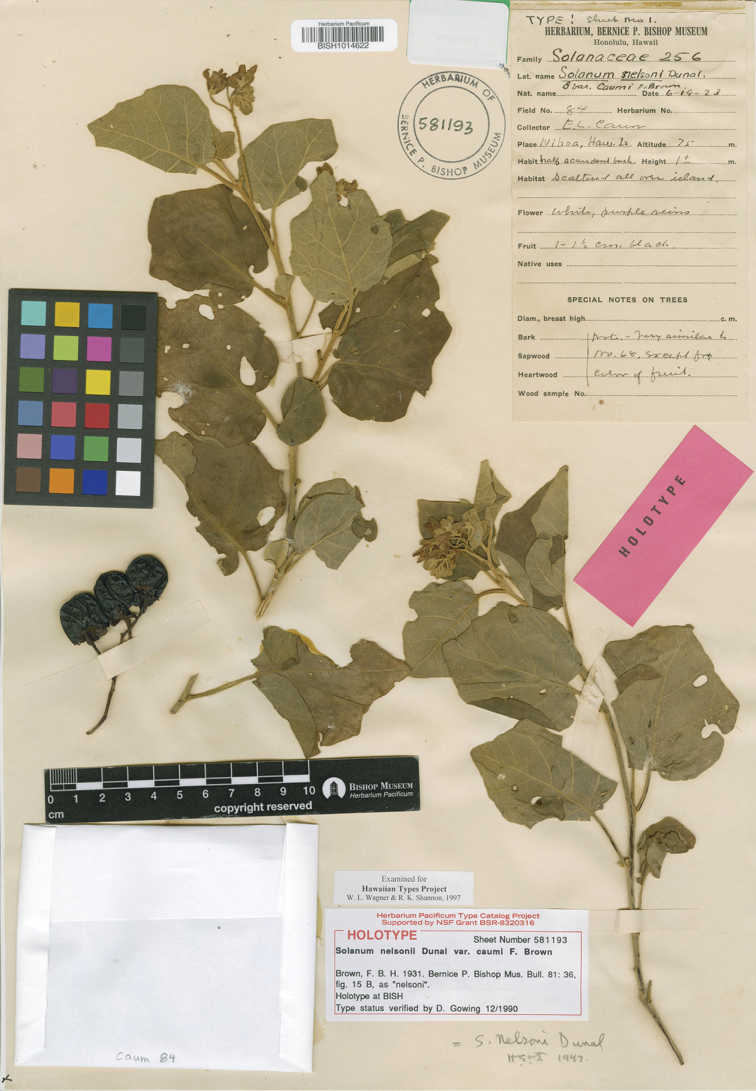
*Solanum caumii* (F.Br.) D.McClelland (holotype of S. nelsonii var. caumii F.Br., *E. Caum 84* BISH [acc. # 581193, barcode BISH1014622]). Reproduced with permission.

##### Description.

Erect to sprawling shrub to ca. 1.5 m, the internodes to 6.6 cm long, unarmed. Stems densely pubescent with yellow-ferruginous, short-stalked porrect-stellate trichomes, the stalks of various lengths to ca. 0.1 mm, the rays 6–10, 0.15–0.2 mm long, the midpoint shorter than the rays; new growth densely stellate-pubescent; bark of older stems reddish brown. Sympodial units difoliate, the leaves geminate or not, if geminate the leaves of a pair similar in size and shape. Leaves simple or shallowly lobed; blades 3.5–8.0 cm long, 2.2–6.4 cm wide, 1.3–1.6 times as long as wide, deltate to ovate, chartaceous, concolorous, unarmed; adaxial surfaces densely pubescent with short-stalked porrect-stellate trichomes, the stalks to ca. 0.1 mm long, the rays 6–9, 0.1–0.2 mm long, the midpoint much shorter than the rays; abaxial surfaces densely pubescent with short-stalked porrect-stellate trichomes, the stalks to ca. 0.1 mm long, the rays 7–9, 0.2–0.25 mm long, the midpoint of the stellate trichomes much shorter than the rays; principal veins 4–6 pairs, the midrib raised abaxially, distinct adaxially, the lateral veins weakly brochidodromous or semicraspedodromous, raised abaxially, distinct adaxially; base cordate, truncate, or rounded; margins entire or shallowly lobed, the sinuses less than 1/4 of the way to the midrib; apex acute to acuminate; petiole 0.8–3.3 cm long, 1.0–1.4 mm in diameter, channeled adaxially, densely pubescent with stalked porrect-stellate trichomes like the stems and leaves. Inflorescence to 8.1 cm long in flower, appearing lateral, extra-axillary, emerging from the distal 1/3 of the internode, typically unbranched but occasionally forked, with 10–16 flowers, densely pubescent with short-stalked porrect-stellate trichomes like the stems and leaves; peduncle 0.5–1.7 cm long; pedicels 0.9–1.2 cm long, 0.3–0.6 mm in diameter at the base, 0.5–0.9 mm in diameter below the calyx, straight, densely pubescent with porrect-stellate trichomes like those of the inflorescence axes, articulated at the base; pedicel scars evenly spaced to 8.8 mm apart, in two rows. Buds globose, the calyx densely stellate-pubescent, the corolla densely stellate-pubescent abaxially where exposed, strongly exerted from the calyx before anthesis. Flowers 4–5-merous, heterostylous, with short-styled flowers borne distally, the plants weakly andromonoecious. Calyx 3.1–4.9 mm long, the tube 1.7–1.8 mm long, the lobes 1.1–3.5 mm long, 1.2–2.1 mm wide, deltate, splitting in the sinuses during fruit development and then the lobes long-triangular, densely pubescent abaxially and adaxially. Corolla 1.3–2.0 cm in diameter, stellate, white, the interpetalar tissue well-developed, glabrous, the petal midveins purple, the lobes 5.1–5.7 mm long, 7.2–7.4 mm wide, deltate, spreading at anthesis, moderately pubescent abaxially, more or less glabrous adaxially. Stamens equal; filament tube minute; free portion of the filaments ca. 1.5 mm long, glabrous; anthers 2.9–3.2 mm long, 0.8–0.9 mm wide, tapering, strongly arcuate and spreading, purple sometimes appearing almost black, poricidal at the tips, the pores directed distally, not splitting with age. Ovary ca. 0.8 mm in diameter, ovate, white or cream in live plants, densely pubescent with porrect-stellate trichomes; styles of long-styled flowers ca. 3.8 mm long, ca. 0.4 mm in diameter, exserted from the anther cone, filiform, straight, densely stellate pubescent in the basal 2/3, in short-styled flowers 2.1–2.5 mm long, 0.1–0.2 mm in diameter, included in the anther cone; stigma ca. 0.3 mm in diameter, capitate, white or cream (live plants). Fruit a globose berry, 1.0–1.5 cm in diameter, green when immature, red or black when mature, juicy, glabrous, the pericarp thin, matte to slightly glossy, opaque; fruiting pedicels 1.1–1.9 cm long, 0.9–1.1 mm in diameter at the base, 2.1–2.3 mm in diameter below the calyx, straight and spreading; fruiting calyx lobes 3.4–6.3 mm long, 1.8–2.5 mm wide, moderately to densely pubescent, appressed to the berry surface. Seeds 20–30 per fruit, 2.8–3.5 mm long, 3.5–4.1 mm wide, flattened-reniform, red-brown when dry, the surface minutely pitted and evenly reticulate, the testal cells straight-sided. Chromosome number not known.

##### Distribution and ecology

**Figure 5.**
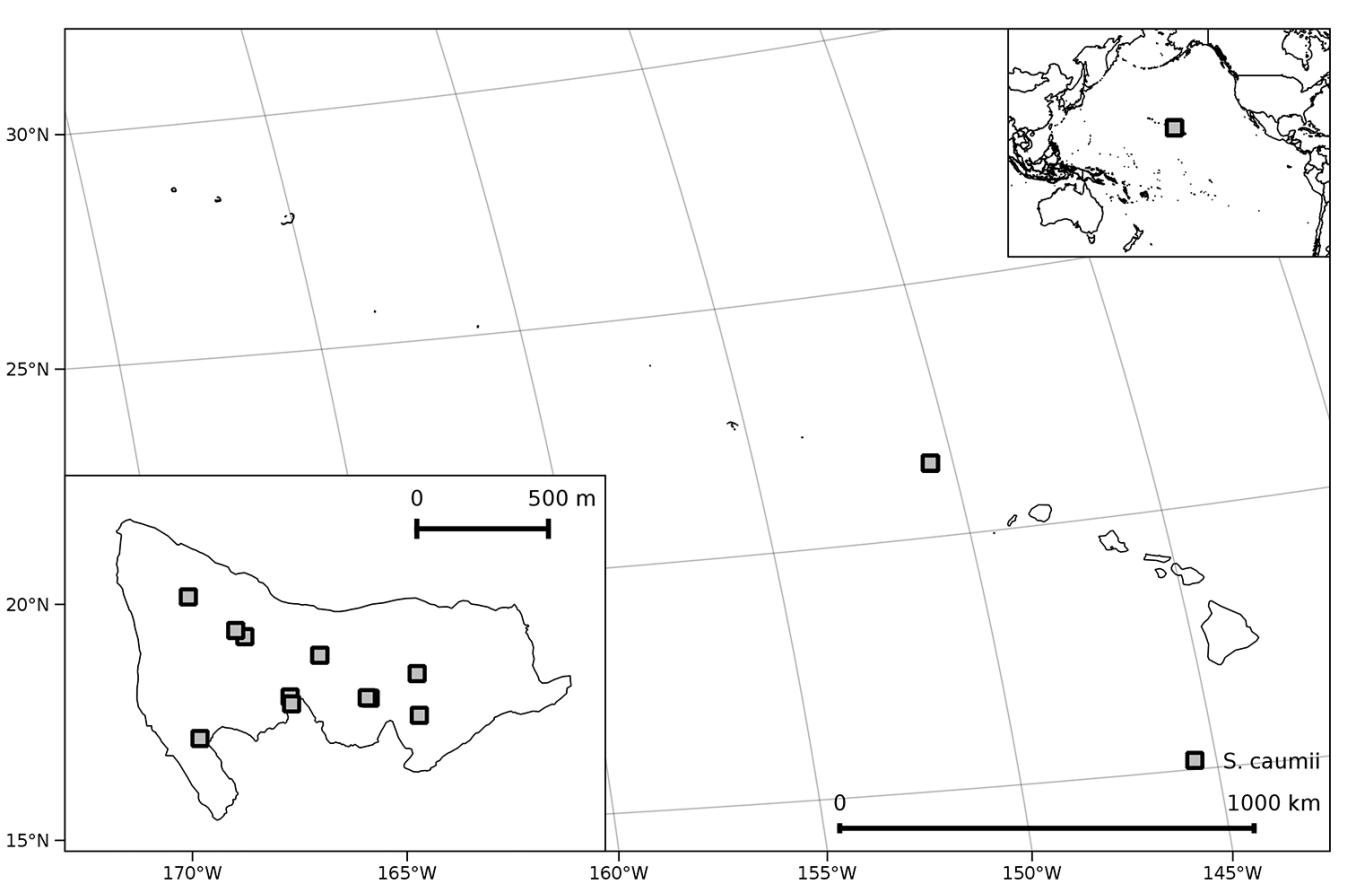
Distribution of *Solanum caumii*.

(Figure 5). *Solanum caumii* is restricted to the state of Hawaii (United States of America) on the tiny island of Nihoa in shallow rocky soil from 10 to 270 m elevation. Plants have been collected from both coastal and higher elevation areas on the island.

##### Phenology.

Known to flower March–April and June–September and fruit March and June–July.

##### Common names and uses.

United States of America. Hawaii (Nihoa): Akia (*St. John 22731*).

##### Etymology.

The epithet honors Edward Leonard Caum (1893–1952), the American botanist who collected the type.

##### Preliminary conservation assessment

(IUCN 2019). EOO = 0.25 km^2^ [CR – Critically Endangered]; AOO = 4 km^2^ [CR – Critically Endangered]. We assess *Solanum caumii* as CR (Critically Endangered) using IUCN Criteria of B1,2a,b; populations may be secure on the isolated island of Nihoa that is largely protected, but no recent studies on population stability have been undertaken. Recognition of *S. caumii* at the species level has conservation consequences. Symon (1999) included *S. caumii* within *S. nelsonii*. At that time *S. nelsonii* was listed as vulnerable; segregation of *S. caumii* the species may change the status of *S. nelsonii*. Threats to *S. caumii* include habitat destruction from natural causes and herbivory by an introduced grasshopper (Mitchell et al. 2005).

##### Discussion.

Brown (1931) recognized the entity we here delimit as *S. caumii* as two varieties of *S. nelsonii*, a coastal species found on many islands in the Hawaiian archipelago. St. John (1988) placed S. nelsonii var. acuminatum in synonymy with S. nelsonii var. caumii. *Solanum caumii* is most similar to *S. nelsonii* but it can be distinguished by its semi-erect habit, larger leaf length to width ratio, and sometimes red fruit. In 2008, plants of *S. nelsonii* s.s. from Molokai were cultivated alongside individuals from Nihoa (*S. caumii*) in greenhouses at the New York Botanical Garden (made available through the conservation program at the National Tropical Botanical Garden in Honolulu by Drs. David and Lida Burney and their staff). Under identical growing conditions plants here recognized as *S. caumii* and *S. nelsonii* s.s. maintained their distinct forms.

Endemism on the small island of Nihoa is relatively common, it is only one of four of the outer Hawaiian islands that still has exposed basalt substrate. There are other species of endemic or near-endemic plants (*Amaranthus brownii* Christoph. & Caum, Amaranthaceae; *Pritchardia remota* Becc., Arecaceae; *Scheidea verticillata* F.Br., Caryophyllaceae) and the entire island has been designated critical habitat for them (Mitchell et al. 2005). The island also hosts two endemic birds, at least seventeen endemic arthropods, and six endemic land snails (Mitchell et al. 2005).

##### Additional specimens examined.

United States of America. Hawaii. Nihoa: sin. loc., 13 Jun 1923 (fl), *Bryan 3* (BISH); Miller Valley, 22 Apr 1983 (fl), *Conant 102* (BISH); base of Miller Valley, ca. 30 ft, 26 Aug 1968 (fl), *Herbst 1210* (BISH, US); Middle Valley, ca. 500 ft, 27 Jul 1980 (fl, fr), *Herbst & Takeuchi 6544* (US); East Palm Valley, ca. 300 ft, 27 Jul 1980 (fl, fr), *Herbst & Takeuchi 6555* (BISH; US); (fr), *Judd s.n.* (K); Rocky Gulch, 100 m, 20 Jun 1923 (fr), *Judd 6* (BISH); Rocky Gulch, 100 m, 20 Jun 1923 (fr), *Judd 7* (BISH); Rocky Gulch, 100 m, 20 Jun 1923 (fl), *Judd 8* (BISH); lower slopes above the east cove, 23 Sep 1964 (fl), *Long 2424* (US); top of Miller’s Peak in crevice at top of “Devil’s Slide”, north side of peak, 24 Sep 1964 (fl), *Long 2434* (US); lower slopes west valley near water-course, ca. 350 ft, 24 Sep 1964 (fl), *Long 2439* (US); near helicopter landing, 6 Mar 1964 (fl, fr), *Munro s.n.* (BISH); Kawaewae, 75 ft, 12 Aug 1947 (fl), *St. John 22731* (K, PTBG, RSA, US); Papahanaumokuakea Marine National Monument, above Miller Valley W facing Miller Ridge, 76 m (fl, fr), 7 Apr 2006 *Tangalin 717* (US); Papahanaumokuakea Marine National Monument, lower Miller Valley just above sea shelf, near National Wildlife Refuge sign and base camp, 12 m, 7 Apr 2006 (fl), *Tangalin 722* (US).

#### Solanum memaoyanum

Taxon classificationPlantaeSolanalesSolanaceae

D. McClelland sp. nov.

3C3B8DE3-DE8B-5002-A557-27C45B79BE36

urn:lsid:ipni.org:names:77209327-1

Figure 6

##### Diagnosis.

Like *Solanum semisucculentum* D.McClelland, but growing in the forest, a taller plant and with pubescent stems, leaves, and inflorescences.

**Figure 6.**
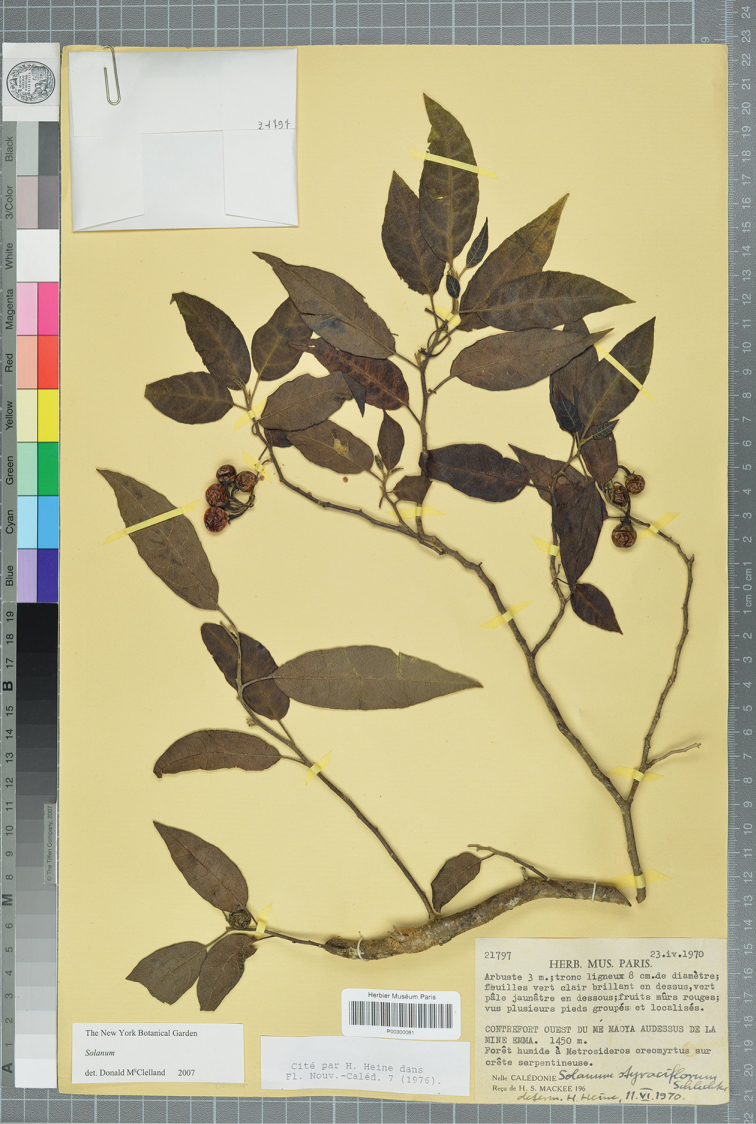
*Solanum memoayanum* D.McClelland (isotype *MacKee 21797*, P [P00300081]). Reproduced with permission.

##### Type.

New Caledonia. Nord: Contrefort ouest du Mé Maoya audessus de la Mine Emma, 1450 m, 23 Apr 1970 (fr), *H.S. MacKee 21797* (holotype: NOU [acc. #017079]; isotypes: A, K [K000922612, K001155402], L [L0651810, L0651811], MO [acc. #2816917, barcode MO-2282963], NSW [acc. #594231], P [P00300081], U [U0182899]).

Shrub or small tree to 3 m tall with a trunk to 8 cm in diameter, the internodes to 2.8 cm long, unarmed. Stems densely pubescent with yellow-ferruginous, sessile porrect-stellate trichomes, the rays ca. 8, 0.05–0.1.5 mm long, the midpoint shorter than to more or less equal to the rays; new growth densely pubescent with sessile porrect-stellate trichomes on the veins and sparsely pubescent on the lamina, with minute glandular papillae to 0.04 mm long; bark of older stems brown, glabrescent. Sympodial units difoliate, the leaves not geminate. Leaves simple; blades 3.9–9.9 cm long, 2.0–3.3 cm wide, ca. 2.0–3.4 times as long as wide, lanceolate to elliptic, somewhat fleshy, discolorous, unarmed; adaxial surfaces sparsely pubescent, later glabrescent, the sessile porrect-stellate trichomes with (4–)8(–12) rays, 0.1–0.25 mm long, the midpoints shorter than to more or less equal to the rays; abaxial surfaces sparsely pubescent with sessile porrect-stellate trichomes, the rays 6–8, 0.1–0.2 mm long, the midpoint shorter than to more or less equal to the rays; principal veins 5–8 pairs, the midrib raised abaxially, distinct adaxially, the lateral veins weakly brochidodromous, raised abaxially, distinct adaxially; base rounded, equilateral or oblique; margins entire; apex acute to short-acuminate; petiole 1.0–2.2 cm long, 0.7–1.2 mm in diameter, channeled above, densely pubescent with sessile porrect-stellate trichomes like the stems. Inflorescence to 2.2 cm long, appearing lateral, extra-axillary, emerging from the upper 1/3 of the internode, unbranched, with 2–7 flowers, densely pubescent with sessile porrect-stellate trichomes; peduncle 0.8–1.7 cm long, 0.5–0.6 mm in diameter, unarmed; pedicels 0.7–1.3 cm long, 0.3–0.4 mm in diameter at the base, 0.6–0.9 mm in diameter below the calyx, bent to approximately 90° below the calyx, gradually increasing in diameter in the distal 1/4–1/2, sparsely pubescent, articulated at the base; pedicel scars congested to spaced 3.5 mm apart, rigid, in two rows. Buds ovate, the calyx densely stellate-pubescent, the lobe tips glabrous, the corolla densely stellate-pubescent abaxially where exposed in bud, strongly exserted from the calyx before anthesis. Flowers 5-merous, all perfect or apparently so (few flowering specimens have been collected). Calyx ca. 2.6 mm long, appearing nearly truncate with caudate lobe tips, the tube ca. 0.9 mm long, the lobes 1.4–1.7 mm long, the sinuses opaque when dry; splitting in the sinuses during fruit development and then the lobes deltate. Corolla ca. 2.4 cm in diameter, stellate, white, the interpetalar tissue well-developed, glabrous, the lobes ca. 0.8 cm long, 0.3 cm wide, triangular, spreading at anthesis, abaxially moderately pubescent, adaxially glabrous at the base with scattered pubescence towards the tips. Stamens equal; filament tube minute; free portion of the filaments ca. 1.7 mm long, glabrous; anthers ca. 5.1 mm long, 1.3 mm wide, tapering, straight, yellow, connivent, poricidal at the tips, the pores directed outward, extending around the tip edge. Ovary ca. 1.3 mm in diameter, globose, glabrous; style and stigma not seen. Fruit a globose juicy berry, 0.7–1.1 cm in diameter, red when mature, glabrous, the pericarp thin, glossy, translucent; fruiting calyx lobes 2.6–4.0 mm long, 1.7–3.8 mm wide, glabrous, reflexed; fruiting pedicels 1.3–2.6 cm long, 0.6–1.0 mm in diameter at the base, 1.7–2.5 mm in diameter below the calyx, gradually increasing in diameter in the distal 1/3–2/3, arching, glabrous to sparsely pubescent. Seeds 40–50 per fruit, 1.8–2.3 mm long, 2.2–2.6 mm wide, flattened-orbicular and notched at the point of attachment, yellow-tan when dry, the surface minutely and deeply pitted (cancellate), the testal cells somewhat sinuate in outline in the center, more straight-sided along the incrassate margins. Chromosome number not known.

##### Distribution and ecology

**Figure 7.**
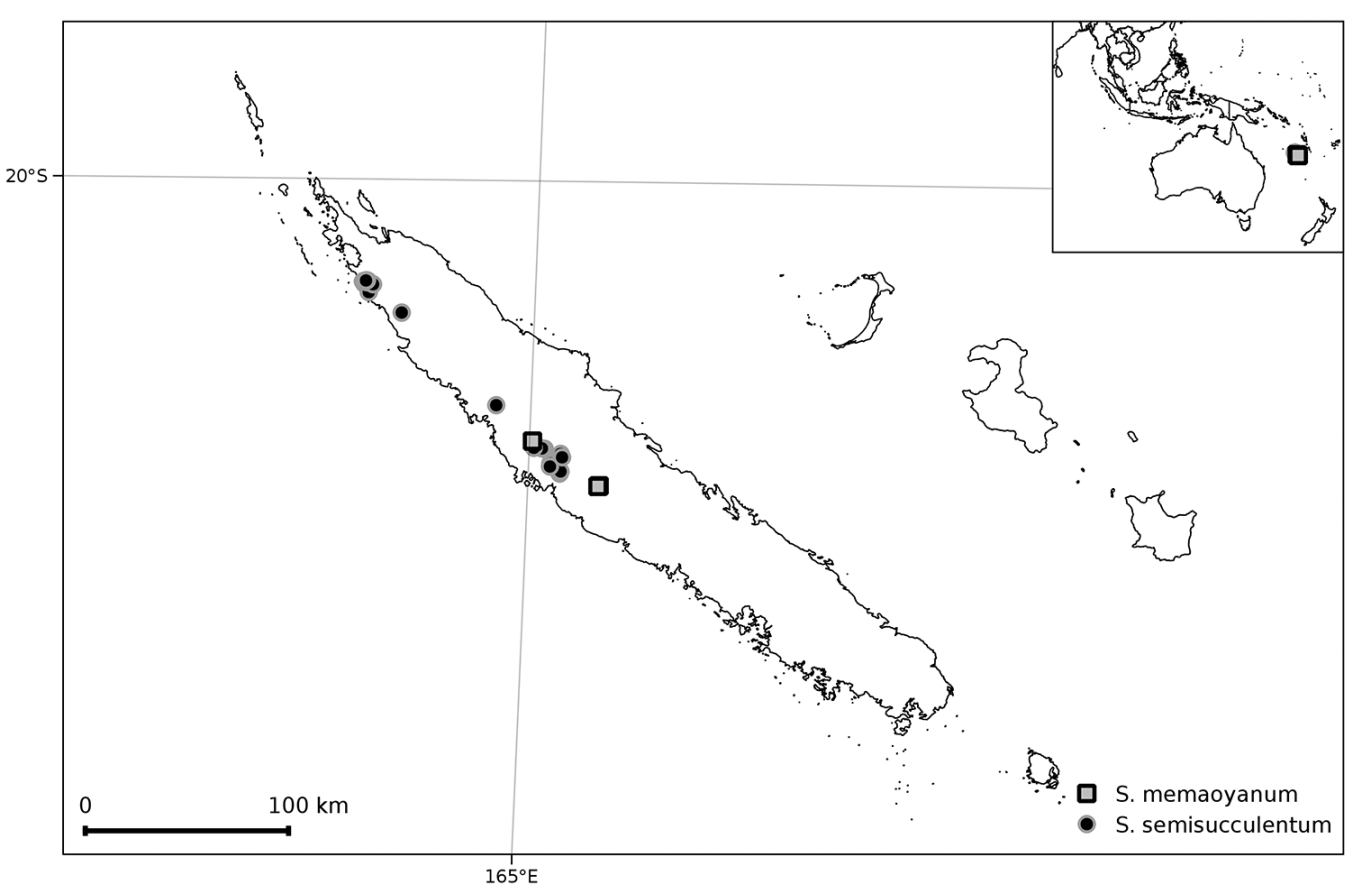
Distribution of *Solanum memoayoanum* and *S. semisucculentum*.

(Figure 7). *Solanum memaoyanum* is narrowly restricted to the mountain of Mé Maoya on the Grande Terre, New Caledonia, and grows in rainforest from 1,300 to 1,500 m elevation.

##### Phenology.

Known to flower in January and fruit in April and August.

##### Etymology.

This species is named after Mé Maoya, the mountain where the type was collected.

##### Preliminary conservation assessment

(IUCN 2019). EOO = 9.35 km^2^ [CR – Critically Endangered]; AOO = 12 km^2^ [EN –Endangered]. *Solanum memaoyanum* is known from only a few collections, all of which are in montane serpentine soil areas now highly disturbed by mining activities. Although the AOO suggests an assessment of EN, we suggest *S. memaoyanum* should be assigned a status of CR (B2a,b) due to its fragmented distribution and threats to its montane habitat.

##### Discussion.

*Solanum memaoyanum* is most similar to another new species described here, *S. semisucculentum*. Specimens here attributed to both were included in Heine’s (1976) concept of *S. styraciflorum* Schltr.; McClelland (2012) treats *S. styraciflorum* as a synonym of *S. artense* Montroux (see description of *S. artense* on Solanaceae Source, www.solanaceaesource.org). From herbarium material *S. memayoanum* appears to have fleshy leaves like *S. semisucculentum*, but it differs in having a taller habit (*MacKee 21797* records the stems as being 8 cm in diameter), pubescent stems, leaves, and inflorescences, and seeds with a more deeply pitted (the lateral cell walls longer) surface. In addition, the habitat of the two species differs; *Solanum memaoyanum* grows in closed rainforest from 1,300 to 1,500 m elevation as opposed to *S. semisucculentum*, which grows in open shrubby habitat (maquis) and is typically not found above 700 m elevation. *Solanum artense* (incl. plants matching the protologue of *S. styraciflorum*) is found below 250 m on calcareous soils, rather than the serpentine soils in mountainous regions on which both *S. memaoyanum* and *S. semisucculentum* are found.

##### Additional specimens examined.

New Caledonia. Nord: contrefort oest du Mé Maoya audessus de la Mine Emma, 1350–1500 m, 13 Jan 1970 (fl), *H.S. MacKee 21422* (NOU, P); Mt. Mé Maoya, above Mine Emma, ca 27 air-km northwest of Bourail, ca. 1300 m, 8 Aug 1980 (fr), *G. McPherson 2948* (MO, NOU, NSW, PTBG).

#### Solanum pseudopedunculatum

Taxon classificationPlantaeSolanalesSolanaceae

D.McClelland sp. nov.

0112C642-3BE6-54A7-B284-614299DC4F5F

urn:lsid:ipni.org:names:77209328-1

Figure 8

##### Diagnosis.

Like *Solanum inamoenum* Benth. but differing in the congested inflorescence with the basal flower borne at the very base of the inflorescence and the inflorescence with a pseudo-peduncle, longer pedicels, and larger flowers and fruits.

**Figure 8.**
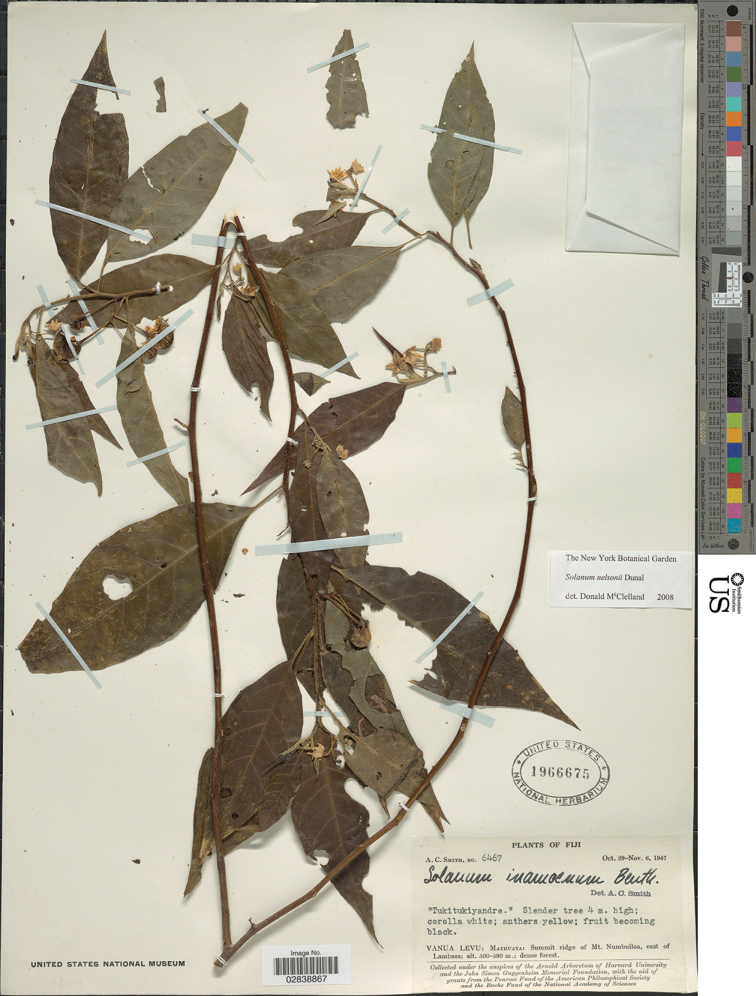
*Solanum pseudopedunculatum* D.McClelland (holotype *Smith 6467*, incorrectly labelled as *S. nelsonii*. US [acc. # 1966675, barcode 02838867]). Reproduced with permission.

##### Type.

Fiji. Vanua Levu: Mathuata, summit ridge of Mt. Numbuiloa, east of Lambasa, 500–590 m, 29 Oct – 6 Nov 1947 (fl, fr), *A.C. Smith 6467* (holotype: US [acc. #1966675, barcode 02838867]; isotypes: A, BISH [acc. #181936], K [K000922644], L [L0531787], NY [00828289], P [P00315297], S).

##### Description.

Shrub or small tree to 4 m, the internodes to 6.5 cm long, unarmed. Stems densely pubescent with yellow, sessile or stalked porrect-stellate trichomes, the stalks to ca. 0.2 mm, the rays 4–8, 0.15–0.2 mm long, the midpoint shorter than the rays; new growth moderately pubescent adaxially with sessile and stalked porrect-stellate trichomes and minute glandular papillae ca. 0.05 mm long; bark reddish brown. Sympodial units difoliate, the leaves geminate, members of a pair more or less equal in size and shape. Leaves simple; blades 6.0–15.0 cm long, 2.0–4.7 cm wide, ca. 2.5–4.2 times as long as wide, ovate to elliptic, chartaceous, concolorous, unarmed; adaxial surfaces glabrescent with scattered stalked porrect-stellate trichomes, the stalks to ca. 0.2 mm long, the rays 4–8, 0.2–0.4 mm long, the midpoint equal to or longer than the rays; abaxially sparsely to moderately pubescent with stalked porrect-stellate trichomes, the stalks to ca. 0.2 mm long, the rays 4–8, 0.25–0.4 mm long, the midpoint equal to or longer than the rays; principal veins 6–8 pairs, the midrib raised abaxially, distinct adaxially, the lateral veins weakly brochidodromous, raised abaxially, distinct adaxially; base cuneate to attentuate, equilateral or oblique; margins entire; apex acuminate, occasionally obtuse; petiole 0.8–2.0 cm long, 0.5–1.0 mm in diameter, moderately stellate-pubescent with trichomes like those of the leaves, channeled above. Inflorescence to ca. 1.3 cm long, appearing lateral, extra-axillary, in the middle 1/3 of the internode, unbranched, with few to 8 flowers, densely pubescent with sessile and stalked porrect-stellate trichomes; peduncle ca. 0.6 cm long, ca. 0.5 mm in diameter, stellate-pubescent like the inflorescence axes; pedicels 0.8–1.4 cm long, 0.4–0.5 mm in diameter at the base, 0.8–1.4 mm in diameter below the calyx, straight, gradually increasing in diameter in the distal 1/4–1/3, moderately to densely pubescent, articulated at the base; pedicel scars evenly spaced 1.0–2.4 mm apart, rigid, in two rows. Buds globose, the calyx moderately stellate-pubescent, the corolla densely stellate-pubescent where exposed in bud, strongly exserted from the calyx before anthesis. Flowers 5-merous, all perfect or apparently so. Calyx 1.0–1.6 mm long, appearing nearly truncate with minute deltate or apiculate lobe tips, the tube 0.4–0.8 mm long, the lobes 0.5–0.8 mm long, not splitting or splitting in the sinuses during fruit development and then the lobes deltate, sparsely to moderately pubescent abaxially, glabrous adaxially. Corolla ca. 1.5 cm in diameter, stellate, white, the interpetalar tissue poorly developed, glabrous, the lobes 3.1–4.2 mm long, 2.2–2.9 mm wide, deltate, spreading at anthesis, glabrous adaxially, moderately stellate-pubescent abaxially. Stamens equal; filament tube minute; free portion of the filaments 0.6–0.9 mm long, glabrous; anthers 1.5–2.1 mm long, 1.0–1.2 mm wide, somewhat tapering, straight, yellow, spreading, poricidal at the tips, the pores directed distally. Ovary ca. 1.2 mm in diameter, globose, with a few stellate hairs and a few minute and simple glandular hairs near the apex; style 3.1–4.0 mm long, 0.3–0.4 mm in diameter, longer than the stamens, exserted beyond the anther cone, filiform, straight, sparsely pubescent in the basal ca. 1/3 with minute glandular hairs; stigma 0.5–0.6 mm in diameter, capitate, minutely papillose. Fruit a globose juicy berry, 0.7–1.0 cm in diameter, variously reported as orange, red becoming black, and purple when mature, glabrous, the pericarp thin, glossy, somewhat translucent; fruiting calyx lobes 0.5–0.9 mm long, 1.5–1.7 mm side, sparsely stellate-pubescent, appressed to the surface of the berry; fruiting pedicels 1.7–2.3 cm long, 0.3–0.6 mm in diameter at the base, 1.1–1.6 mm in diameter below the calyx, straight, gradually increasing in diameter in the distal 1/3–1/2, sparsely stellate-pubescent. Seeds 20–40 per fruit, 1.8–2.0 mm long, 2.3–2.8 mm wide, flattened-orbicular and notched at the point of attachment to flattened-reniform, yellow-tan when dry, the surface with the central area nearly smooth, the margins minutely pitted with the testal cells straight-sided (alveolate). Chromosome number not known.

##### Distribution and ecology

**Figure 9.**
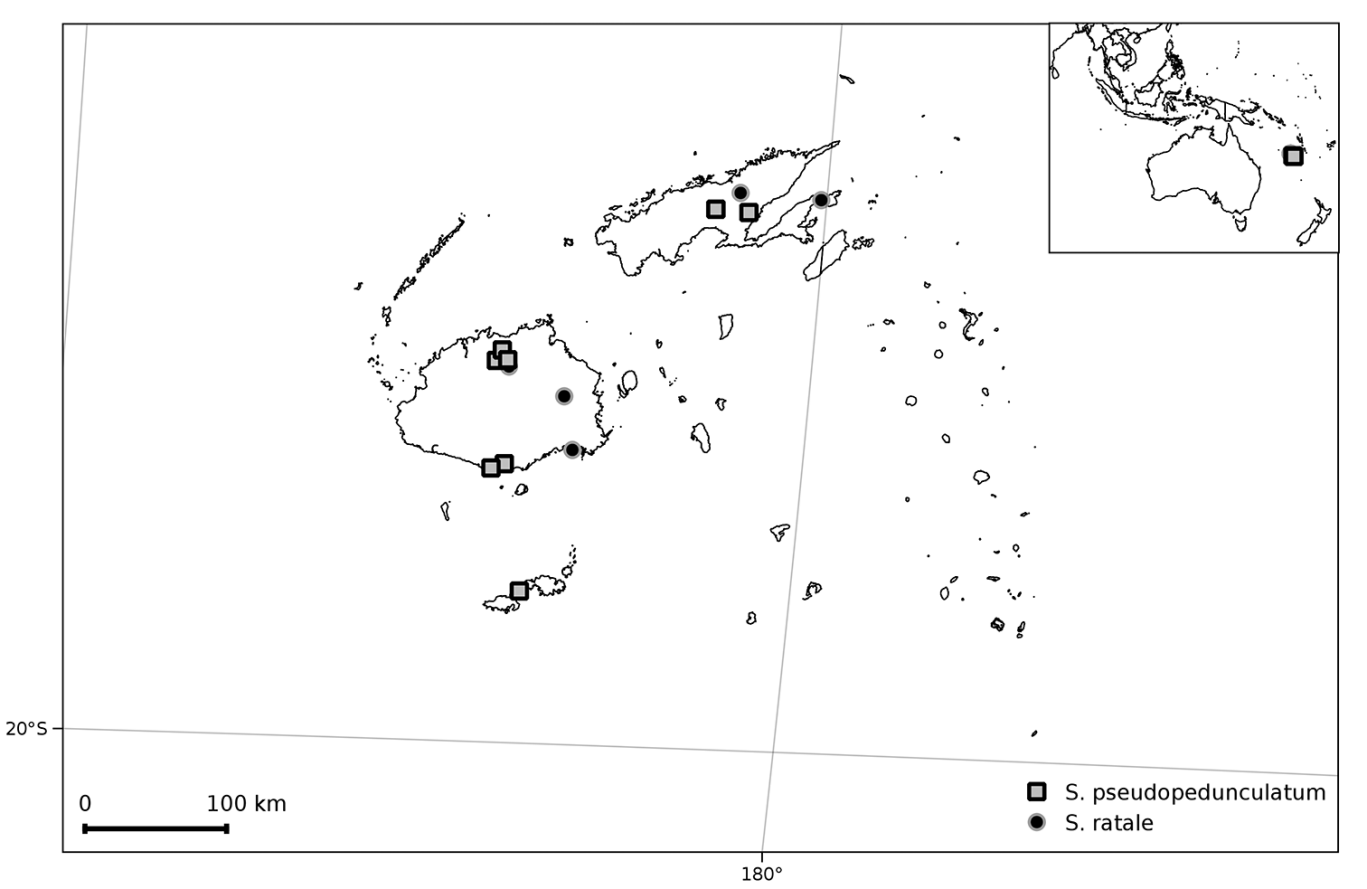
Distribution of *Solanum pseudopedunculatum* and *S. ratale*.

(Figure 9). *Solanum pseudopedunculatum* is endemic to Fiji on the islands of Kanduvau, Vanua Levu, and Viti Levu; it grows in forest and secondary thickets, from 50 to 1,150 m elevation.

##### Phenology.

Known to flower and fruit February–March and July–December.

##### Common names and uses.

Fiji. Moloa (*Smith 603*); tukitukiyandre (*Smith 6467*).

##### Etymology.

The specific epithet is derived from the “pseudo-peduncle” of the inflorescence of this species, where the space between the lowermost flower and the rest gives the appearance of an elongate peduncle.

##### Preliminary conservation assessment

(IUCN 2019). EOO = 17,482 km^2^ [VU – Vulnerable]; AOO = 24 km^2^ [EN –Endangered]. Based on the paucity of recent collections and the fragmented populations (5 locations) on three of the islands of the Fijian archipelago we assess *Solanum pseudopedunculatum* as VU (B1a,b D2).

##### Discussion.

In his Flora of Fiji, Smith (1991) included many of the specimens here recognized as *S. pseudopedunculatum* in his concept of *S. inamoenum* Benth. The two species are not very similar morphologically, however, with leaf and stem pubescence differing in density (*S. inamoenum* pubescence is denser and the trichome rays are somewhat longer than in *S. pseudopedunculatum*), inflorescence structure (unbranched in *S. pseudopedunculatum* and highly branched in *S. inamoenum*), flower size (to 1 cm in diameter and homostylous in S. *pseudopedunculatum*, larger and heterostylous in *S. inamoenum*) and habitat preference (*S. pseudopedunculatum* is a plant of forests and secondary thickets, while *S. inamoenum* grows near the coast). *Solanum pseudopedunculatum* is much more similar to *S. ratale* but can be distinguished by its taller habit, mature leaves that retain pubescence along the veins and lamina, longer pedicels, and larger corolla, anthers, and berries.

##### Specimens examined.

Fiji. sin. loc., Dec 1904-Mar 1905 (fr), *Goddard s.n.* (NSW). Kandavu: Hills above Namalata and Ngaloa Bays, 200–400 m, 13–18 Oct 1933 (fl, fr), *Smith 141* (BISH, GH, K, NY, P, S, UC, US). Vanua Levu: Mt. Delaikoro, Macuata, 3050 ft, 21 Aug 1962 (fl, fr), *Parham & Koroi 12792* (BISH); Thakaundrove, SW slope of Mt. Mbatini [Batini], 300–700 m, 28–29 Nov 1933 (fl, fr), *Smith 603* (GH, K, NY, P, S, UC, US). Viti Levu: Serua district, 500 ft, 14 Jun 1961 (fl, fr), *Bola 42* (K); Mba [“Tholo North”], Sovutawambu, near Nandrivatu, 750–800 m, 27 Feb – 4 Mar 1941 (fl, fr), *Degener 14594* (A, K, NY, US); Nadarivatu, 2700 ft, 1907 (fl, fr), *Gibbs 615* (BM×2); Mba (formerly Tholo North), western and southern slopes of Mt. Tomanivi (Mt. Victoria), 850–1150 m, 7 Jul – 18 Sep 1947 (fl, fr), *Smith 5273* (A, K, L, NY, P, S, US); Serua, Hills west of Waivunu Creek, between Ngaloa and Korovou, 50–150 m, 23 Nov – 7 Dec 1953 (fl, fr), *Smith 9223* (BISH, GH, K, L, NY, P, S, UC, US); Nadarivatu, road to Suva, 27 Nov 1906 (fl, fr), *Thurn 294* (K).

#### Solanum ratale

Taxon classificationPlantaeSolanalesSolanaceae

D. McClelland sp. nov.

31773F21-637B-572D-AD11-7CC7421699A7

urn:lsid:ipni.org:names:77209329-1

Figure 10

##### Diagnosis.

Like *Solanum pseudopedunculatum* D.McClelland but differing in shorter stature, densely glandular new growth, stellate trichomes with elongate midpoints that sometimes lack rays, smaller flowers and smaller, few-seeded fruits.

**Figure 10.**
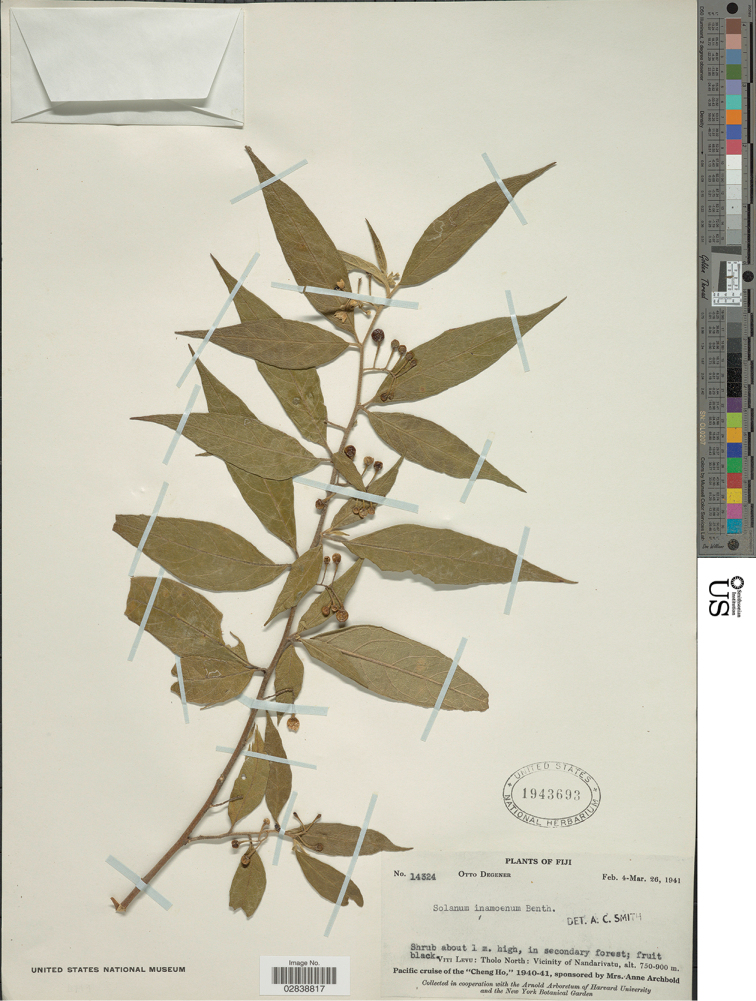
*Solanum ratale* D.McClelland (isotype *Degener 14324*, US [acc. # 1943693, barcode 02838817]).

##### Type.

Fiji. Viti Levu: Mba, (Tholo North), Western division, village of Nadarivatu, Fish Hatchery 2600 ft, 9 Feb 1941 (fl, fr), *O. Degener 14324* (holotype: A; isotypes: K [K000922635, K001155453], UC [acc. #1016245], US [acc. #1943693, barcode 02838817])

##### Description.

Shrub or small tree 0.5–1.5 m, the internodes to 3.4 cm long, unarmed or densely armed. Stems densely pubescent with yellow-ferruginous, sessile or short-stalked porrect-stellate trichomes, the stalks of various lengths to 0.2 mm long, somewhat bulbous, the rays 6–8 or absent, 0.15–0.25 mm long, the midpoint to 1.2 mm long, the prickles, if present, 4–6 mm long, dense, straight and somewhat retrorse (downward pointing), yellow or straw-colored; new growth densely to sparsely pubescent with sessile and short-stalked porrect-stellate trichomes and densely pubescent with minute glandular papillae ca. 0.04 mm long; bark of older stems reddish brown. Sympodial units difoliate, the leaves geminate, members of a pair similar in size and shape. Leaves simple; blades 4.3–10.4 cm long, 1.3–2.6 cm wide, 2.8–5.2 times as long as wide, narrowly lanceolate to narrowly elliptic, chartaceous to subcoriaceous, concolorous, armed or unarmed; adaxial surfaces moderately to sparsely pubescent with sessile and very short-stalked porrect-stellate trichomes and in some individuals with scattered prickles 3–4 mm long, along the veins, the stellate trichomes with the stalks to 0.6 mm long, the rays 4–8 or absent and the trichomes appearing simple, 0.2–0.3 mm long, the midpoint 1–2 mm long, sometimes gland-tipped; abaxial surfaces densely pubescent with sessile and stalked porrect-stellate trichomes and sometimes with prickles along the veins, the stellate trichomes with bulbous stalks to 0.1 mm long, the rays 6–8 or absent and the trichomes appearing simple, 0.25–0.4 mm long, the midpoint 1–2 mm long, much longer than the rays, the prickles if present 2–4 mm long, on the principal veins only, not on the midrib; principal veins 5–7 pairs, the midrib raised abaxially, raised adaxially, the lateral veins weakly brochidodromous, raised abaxially, raised adaxially, if the rest of the plant prickly then the veins with scattered, straw-colored prickles; base cuneate or rounded, equilateral or oblique; margins entire; apex acuminate; petiole 0.4–1.3 cm long, 0.6–1.0 mm in diameter, moderately to densely stellate-pubescent and sometimes with a few straight prickles like the leaf surfaces and stems, channeled above. Inflorescence to 1.8 cm long, appearing lateral, extra-axillary, emerging from the middle 1/3 of the internode, usually unbranched, occasionally forked, with (1-)4–13 flowers, moderately to densely pubescent with sessile and stalked porrect-stellate trichomes like those of the stems, in prickly individuals with 1–3 prickles; peduncle to 0.9 cm long, to 0.6 mm in diameter, pubescent and prickly like the stems; pedicels 0.6–0.8 cm long, ca. 0.3 mm in diameter at the base, ca. 0.6 mm in diameter below the calyx, bent to approximately 90° below the calyx, gradually increasing in diameter in the distal ca. 1/3, moderately to densely pubescent, articulated at the base; pedicel scars congested to spaced 2.2 mm apart, rigid, in two rows. Buds globose, the calyx moderately stellate-pubescent, the corolla densely stellate-pubescent where exposed abaxially, strongly exserted from the calyx before anthesis. Flowers 5-merous, all perfect. Calyx 1.5–2.3 mm long, appearing nearly truncate with caudate to subulate lobe tips, the tube 0.6–1.1 mm long, the lobe tips 0.9–1.3 mm long, the sinuses opaque when dry, not splitting or splitting in the sinuses during fruit development and then the lobes deltate, moderately pubescent abaxially, glabrous adaxially. Corolla ca. 0.5 cm in diameter, stellate, white, the interpetalar tissue well-developed, glabrous, the lobes 3.2–3.8 mm long, 1.9–2.2 mm wide, oblong, spreading at anthesis, moderately pubescent abaxially along the midvein, glabrous adaxially. Stamens equal; filament tube minute; free portion of the filaments ca. 0.5 mm long, glabrous; anthers ca. 1.3 mm long, 0.6 mm wide, oblong, somewhat incurved, apparently yellow, spreading, poricidal at the tips, the pores directed distally, extending around the edge of the apex. Ovary ca. 0.8 mm in diameter, globose, with a few simple glandular hairs at the apex; style ca. 2.8 mm long, ca. 0.3 mm in diameter, exserted from the anther cone, cylindrical, straight, glabrous; stigma ca. 0.5 mm in diameter, capitate, the surface minutely papillate. Fruit a globose, apparently juicy berry, 0.5–0.7 cm in diameter, black or deep purple when mature, glabrous, the pericarp thin, glossy, opaque; fruiting calyx not markedly accrescent, the lobes 2–3(-4) mm long, 1.3–1.6 mm wide, sparsely to moderately pubescent, appressed or somewhat reflexed; fruiting pedicels 0.8–1.5 cm long, ca. 0.5 mm in diameter at the base, 1.4–2.0 mm in diameter below the calyx, straight, gradually increasing in diameter in the distal ca. 2/3, sparsely to moderately pubescent,. Seeds 4–12 per berry, 2.3–2.5 mm long, 1.5–1.9 mm wide, flattened-reniform, yellow-tan when dry, the surface with the central area nearly smooth, the margins minutely and quite deeply pitted (alveolate), the walls of the testal cells straight. Chromosome number not known.

##### Distribution and ecology

(Figure 9). *Solanum ratale* is endemic to Fiji on the islands of Viti Levu and Vanua Levu. It is known from elevation of 200 to 800 m but may grow at lower elevation near Suva (but see below). The habitat has been recorded as logged-off forest (*Degener 14324*) or dense forest along streams (*Smith 5370*).

##### Phenology.

Known to flower and fruit February, May, and June.

##### Etymology.

The specific epithet which means “pertaining to rafts” references a note on *Horne 679* indicating that the light wood was used for making rafts. Another specimen bearing Horne’s collection number of 679 (K000687673, *Trichospermum calyculatum* (Seem.) Burret of the Tiliaceae) has a label “on waste land between Suva and the town of Koluba, Viti Levu, June 1878” but makes no mention of uses. This suggests a possible label mix-up, with the original label of *Horne 679* being affixed to the duplicate of *Horne 678*; *Trichospermum* is a small tree with light wood, more likely to be used for making rafts and growing at lower elevations. Horne (1881), however, lists “Solanum sp. nov.” as both his 678 and 679, so it appears he used the number 679 twice.

##### Preliminary conservation assessment

(IUCN 2019). EOO = 862 km^2^ [EN –Endangered]; AOO = 16 km^2^ [EN –Endangered]. *Solanum ratale* is known from only a few locations in forest, and no recent collections. We therefore assess it as EN (B1,2a,b), based on its relatively restricted distribution and the threats to forest habitat in island archipelagoes.

##### Discussion.

Very little is known about *S. ratale* due to the few collections in herbaria. It is most similar to *S. pseudopedunculatum* but can be distinguished by its shorter habit, occasionally prickly individuals, shorter pedicels, and smaller corolla, anthers, and fruit.

*Solanum ratale* is polymorphic for presence of prickles on all parts, as is found in many other spiny solanums, both in the Old and New World (e.g., *S. elaeagnifolium* Cav., see Knapp et al. 2017; *S. setaceum* Dammer and *S. schumannianum* Dammer, see Vorontsova and Knapp 2016). The collection *Smith 5370* from Viti Levu is densely prickly, but otherwise conforms exactly to the other collections of *S. ratale*. This collection has been identified in herbaria as *S. retrorsum* Elmer, a Philippine species with a similar polymorphism in the possession of retrorse prickles, but differing in its plurifoliate sympodial units, larger trichomes with midpoints equal to the rays, and bright red berry.

##### Additional specimens examined.

Fiji. Vanua Levu: Mt. Labasa, 15 Jul 1883, *Greenwood 619* (K); Rabi [Rambi] Island, May 1878 (fl, fr), *Horne 678* (K). Viti Levu: Central Division, near Lutu, May 1878 (fl, fr), *Horne 595* (GH, K); near Suva, Jun 1878 (fl, fr) *Horne 679 [a*] (GH, K); Mba (formerly Tholo North), valley of Nggaliwana Creek, N of sawmill at Navai, 725–850 m, 21 Jul 1947, *Smith 5370* (A, US).

#### Solanum semisucculentum

Taxon classificationPlantaeSolanalesSolanaceae

D. McClelland sp. nov.

A4DC1CEF-9CE1-51D6-AABC-22FA79E6D8C1

urn:lsid:ipni.org:names:77209330-1

Figure 11

##### Diagnosis.

Like *Solanum artense* Montroux, but differing in its high elevation serpentine substrate habitat, very fleshy, semi-succulent stems and leaves, glabrous petioles and larger berries.

**Figure 11.**
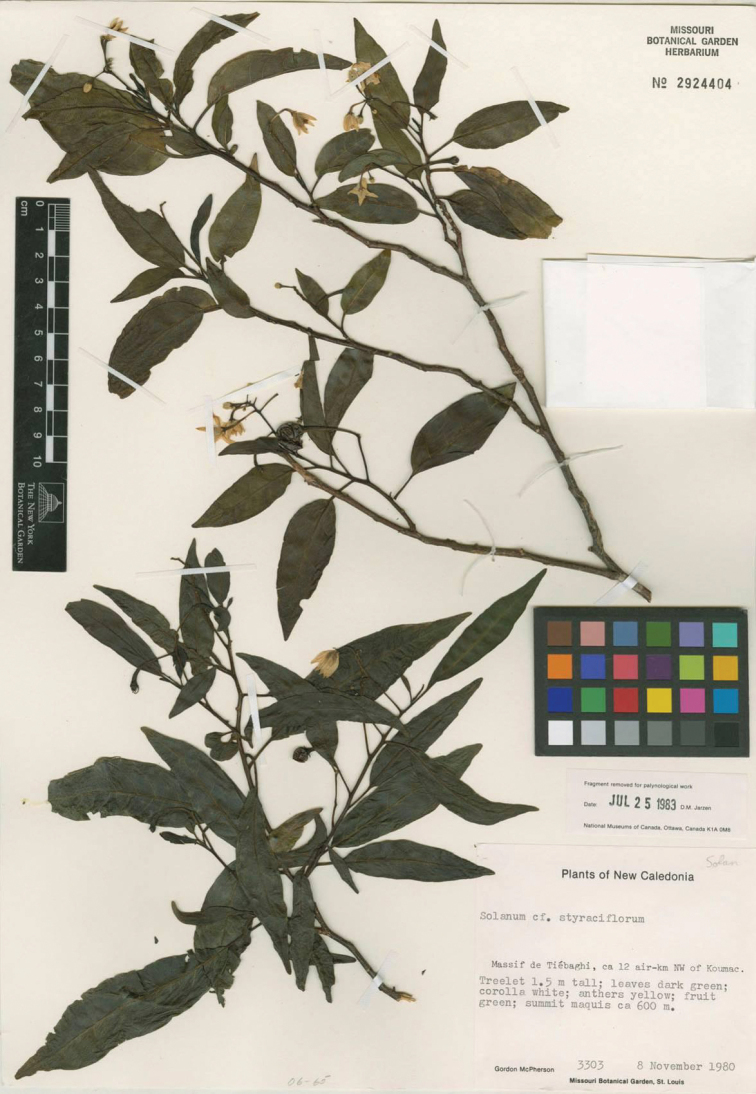
*Solanum semisucculentum* D.McClelland (isotype *McPherson 3303*, MO [acc. # 2924404, barcode MO-2282964]). Reproduced with permission.

##### Type.

New Caledonia. Nord: Tiébaghi Massif, ca. 12 air-km northwest of Koumac, 8 Nov 1980 (fl, fr), *G. McPherson 3303* (holotype: NOU [acc. #017344]; isotypes: MO [acc. #2924404, barcode MO-2282964], NSW [acc. #594228], P [P00301276], PTBG [acc. #032935]).

##### Description.

Shrub 1.5 m, the internodes to 4.5 m long, unarmed. Stems glabrous or with occasional yellow-ferruginous, sessile porrect-stellate trichomes, the rays 7–11, 0.05–0.15 mm long, the midpoint more or less equal to the rays, flexed to lie parallel to the stem, the stellate trichomes soon deciduous; new growth sparsely pubescent with sessile porrect-stellate trichomes and minute glandular hairs to 0.7 mm long; bark of older stems grey, the young stems bright purple. Sympodial units difoliate, the leaves not geminate. Leaves simple; blades 2.3–11.2 cm long, 1.0–3.4 cm wide, 2.3–3.6 times as long as wide, lanceolate to elliptic, fleshy to semisucculent, concolorous; adaxial surfaces glabrous or with a few scattered sessile porrect-stellate trichomes, if present these with 8–10 rays 0.1–0.15 mm long, the midpoint more or less equal to the rays; abaxial surfaces glabrous; principal veins 6–8(–9) pairs, the midrib raised abaxially and adaxially, the lateral veins weakly or strongly brochidodromous, raised abaxially, distinct adaxially; base rounded, cuneate, or short-attenuate, sometimes oblique; margins entire; apex acute to acuminate; petiole 0.6–3.0 cm long, 0.6–0.9 mm in diameter, glabrous or sparsely pubescent on the adaxial surface, channeled above. Inflorescence to 3.8 cm long, appearing lateral, extra-axillary, emerging from the upper 1/3 of the internode, unbranched, with up to 23 flowers, nearly glabrous, with occasional sessile porrect-stellate trichomes and minute glandular papillae; peduncle 1.0–1.4 cm long, 0.4–0.6 mm in diameter; pedicels 0.9–1.2 cm long, 0.2–0.4 mm in diameter at the base, 0.6–0.8 mm in diameter below the calyx, straight, bent approximately 90° below the calyx, gradually increasing in diameter in the distal 1/3–1/2, glabrous, articulated at the base; pedicel scars widely and evenly spaced 5–8 mm apart. Buds ovate, the calyx more or less glabrous to sparsely stellate-pubescent, the corolla densely stellate-pubescent where exposed abaxially, strongly exserted from the calyx before anthesis. Flowers 5(–6)-merous, heterostylous and the plants weakly andromonoecious. Calyx 1.3–5.3 mm long, appearing nearly truncate with apiculate to caudate lobe tips, the tube 0.5–1.1 mm long, the sinuses translucent when dry, the lobes 0.7–4.2 mm long, long-caudate, glabrous adaxially, sparsely pubescent abaxially, splitting in the sinuses during fruit development and then the lobes deltate. Corolla 1.2–2.5 cm in diameter, stellate, white, the interpetalar tissue well developed, the lobes 5.5–8.9 mm long, 5.4–6.8 mm wide, deltate, spreading at anthesis, adaxially glabrous at the base becoming densely stellate pubescent towards the apex, abaxially moderately to densely pubescent. Stamens equal; filament tube minute; free portion of the filaments 0.6–1.0 mm long, glabrous; anthers 4.2–6.2 mm long, 1.3–1.7 mm wide, markedly tapering, straight, yellow, more or less spreading, poricidal at the tips, the pores directed distally, extending around the edge of the apex. Ovary ca. 1.0 mm in diameter, globose, densely stellate-pubescent at the very apex only; style of long-styled flowers 7.7–8.3 mm long, ca. 0.3 mm in diameter, exserted from the anther cone, filiform, straight, moderately to densely pubescent on the basal 1/2–2/3 portion, the style of short-styled flowers 1.0–1.3 mm long, ca. 0.2 mm in diameter, included within the anther cone; stigma 0.4–0.6 mm in diameter, capitate, green in live plants. Fruit a globose or slightly elongate juicy berry, 0.8–1.3 cm in diameter, when immature evenly green, red to orange when mature, glabrous, the pericarp thin, glossy, opaque; fruiting calyx lobes 1.8–2.8 mm long, 0.9–3.5 mm wide, glabrous, appressed to the berry surface or reflexed; fruiting pedicels 1.5–2.4 cm long, 0.6–1.1 mm in diameter at the base, 1.6–2.9 mm in diameter below the calyx, straight or arching, gradually increasing in diameter in the distal 1/2–2/3, glabrous. Seeds 20–30(-40) per berry, ca. 2.2 mm long, 2.3–2.8 mm wide, flattened-orbicular and notched at the point of attachment, red-brown or tan when dry, the surface minutely pitted (alveolate) under a smooth, translucent seed coat, the walls of testal cells straight or slightly sinuate towards the seed center. Chromosome number not known.

##### Distribution and ecology

(Figure 7). *Solanum semisucculentum* is endemic to New Caledonia and is restricted to the ultramafic mountains of the western side of the Grande Terre. It is found in the North Province from (50–)100 to 700(–1,200) m elevation. This species is a serpentine endemic and grows in an open shrubby habitat (maquis) on soils which are fast draining and the semisucculent leaves of this species are possibly an adaptation for these harsh conditions.

##### Phenology.

Flowering and fruiting year round.

##### Etymology.

The specific epithet indicates the texture of the leaves and stems. This texture is best described as fleshy to semisucculent and is unusual in *Solanum*.

##### Preliminary conservation assessment

(IUCN 2019). EOO = 814 km^2^ [EN –Endangered]; AOO = 60 km^2^ [EN –Endangered]. *Solanum semisucculentum* is found in a number of localities in northern New Caledonia, all of which are in montane serpentine soil areas now highly disturbed by mining activities. We preliminarily assign an assessment of EN (B1a,b) due to its fragmented distribution and threats to its montane habitat from mining activities.

##### Discussion.

Heine (1976) treated specimens here recognized as *S. semisucculentum* in his concept of *S. styraciflorum*. The type of *S. styraciflorum* was probably destroyed at B during the Second World War, and no duplicates have been traced; specimens matching the protologue of *S. styraciflorum* exist (see www.solanaceasource.org) and McClelland (2012) treats *S. styraciflorum* as a synonym of *S. artense* Montroux. *Solanum artense* is sparsely to densely pubescent on the stems, along the midvein of the leaves adaxially, and sometimes the inflorescence; its leaves are chartaceous and typically have a greater leaf length to width ratio than *S. semisucculentum*. Specimens of *S. semisucculentum* differ from the protologue of *S. styraciflorum* in petiole pubescence (glabrous or very sparsely pubescent versus densely stellate-subvillose), berry size (7.5–12.5 mm diameter versus ca. 6.0 mm) and elevational range (300–500 m versus 50 m). *Solanum artense* (incl. plants matching the protologue of *S. styraciflorum*) is found below 250 m on calcareous soils while *S. semisucculentum* is typically found at higher elevations, such as the plateau of the Dôme de Tiébaghi, at 300–500 m on lateritic soils.

Perhaps the most remarkable feature of *S. semisucculentum* is its very fleshy, semisucculent leaves. Many species of *Solanum* wilt shortly after collecting; *S. semisucculentum*, however, appears well adapted for water deprivation. With no difficulty, a specimen was kept alive after collecting it with a few roots but no soil and carrying it for a couple hours on a warm tropical afternoon (DHRM, pers. obs.). After a couple of days this specimen opened a flower which had been in bud when it was collected. *Solanum semisucculentum* has vividly purple stems which are quite striking in living material, but dry black on herbarium specimens. This coloration may provide a certain amount of protection of the high levels of solar radiation this species receives in its open maquis habitat.

##### Specimens examined.

New Caledonia. Nord: Dôme de Tiébaghi, 22 Jan 1976 (fl, fr), *Blanchon 1444* (NOU, P); Mine Oubliée [near Pic Poya], 650 m, 7 Jan 1962 (fl, fr), *Catala-Stucki 160* (G); Tiébaghi, 24 Sep 1961 (fl, fr), *Denizot s.n.* (P); Dôme de Tiébaghi, 8 Nov 1980 (fl), *Hoff 2975* (NOU); Mt. Ninga, 1000 m, 15 Oct 1975 (fr), *Jaffré 1415* (NOU, P [date 2 May 1976]); Dôme de Tiébaghi, 350 m, 15 Nov 1975, *Jaffré 1415bis* (P); Plateau de la Tiébaghi, 500 m, 15 Nov 1976 (fl, fr), *Jaffré 1824* (NOU, P); Massif Boulinda, secteur Col Nekoro, 27 Jul 1972 (fl), *Jaffré 2183* (NOU); Massif de Boulinda, above river Ouaha, east of Muéo, 100–200 m, 21.20 S, 165.05 E, 12 Dec 1973 (fl), *Jaffré 19234* (NOU, P); Dôme de Tiébaghi, Pente Sud-Ouest du Dôme de Tiébaghi, 300–500 m, 9 May 1966 (fl), *MacKee 14921* (K, P); pente nord du Mt. Kaala, 400–700 m, 9 Jul 1966 (fl, fr), *MacKee 15272* (A, K, L, NOU, P); pente Nord du Mont Kaala, 700 m, 25 Dec 1966 (fl, fr), *MacKee 16152* (K, L, NOU, NY); Haute Népoui, Oué Péoué, contrefort sud du Kopéto, 500 m, 8 Jul 1970 (fl), *MacKee 22193* (P); Mt. Boulinda, 1200 m, 1 Jun 1972 (fl, fr), *McKee 25571* (P); Dôme de Tiébaghi, 400 m, 30 Nov 1972 (fl, fr), *MacKee 25953* (K, L, P); Nekoro, 200 m, 21 May 1977 (fr), *MacKee 33195* (NOU, P); Dôme de Tiébaghi, Plateau central, 550 m, 8 Jul 1978 (fl, fr), *MacKee 35432* (L, NOU, P); Koumac, Chagrin, 300 m, 8 Jan 1983 (fr), *MacKee 41156* (P); Dôme de Tiébaghi, on the plateau south of the old town, 590 m, 15 Jul 2009 (fl, fr), *McClelland & Nee 554* (MO, NOU, NY, P); Tiébaghi Massif, north of Koumac, 550 m, 20 Dec 1983 (fl, fr), *McPherson 6169* (MO, NOU, PTBG); Boulinda, base, 23 Jan 2008 (fl, fr), *Munzinger et al. 4959* (NOU); Tiébaghi, sur le plateau, côté Est, 14 Apr 2014 (fl, fr), *Munzinger et al. 7575* (NOU); Dôme de la Tiébaghi, 400–600 m, 25 Jul 2007 (fl, fr) *Pillon et al. 775* (NOU); pentes du Dôme de Tiébaghi, 13 Jun 1974 (fl, fr), *Sévenet 685* (NOU); route minière d’accès au Bulinda [Boulinda], 20 Feb 1978 (fl, fr), *Suprin 254* (NOU); Mt. Boulinda, 550 m, 26 Apr 1965 (fr), *Veillon 129* (NOU, P); Dôme de Tiébaghi, plateau, ca. 550 m, 17 Aug 1965 (fl), *Veillon 361* (NOU); Mt. Boulinda, ca. 400 m, 26 Jul 1967 (fl), *Veillon 1268* (K, NOU, P); Dôme de Tiébaghi, ca. 550 m, 25 Nov 1967 (fl, fr), *Veillon 1457* (NOU, P); pentes ouest du Dôme de Tiébaghi, ca. 500 m, 27 Oct 1943 (fl, fr), *Virot 1274* (A, NOU, P).

#### Solanum vanuatuense

Taxon classificationPlantaeSolanalesSolanaceae

D.McClelland sp. nov.

92356232-12A1-51EF-A334-564762956F0C

urn:lsid:ipni.org:names:77209331-1

Figure 12

##### Diagnosis.

Like *Solanum milnei* Seem. and *S. austrocaledonicum* Seem., but differing in usually geminate, lobed leaves, smaller corolla and anthers, and strongly curved style.

**Figure 12.**
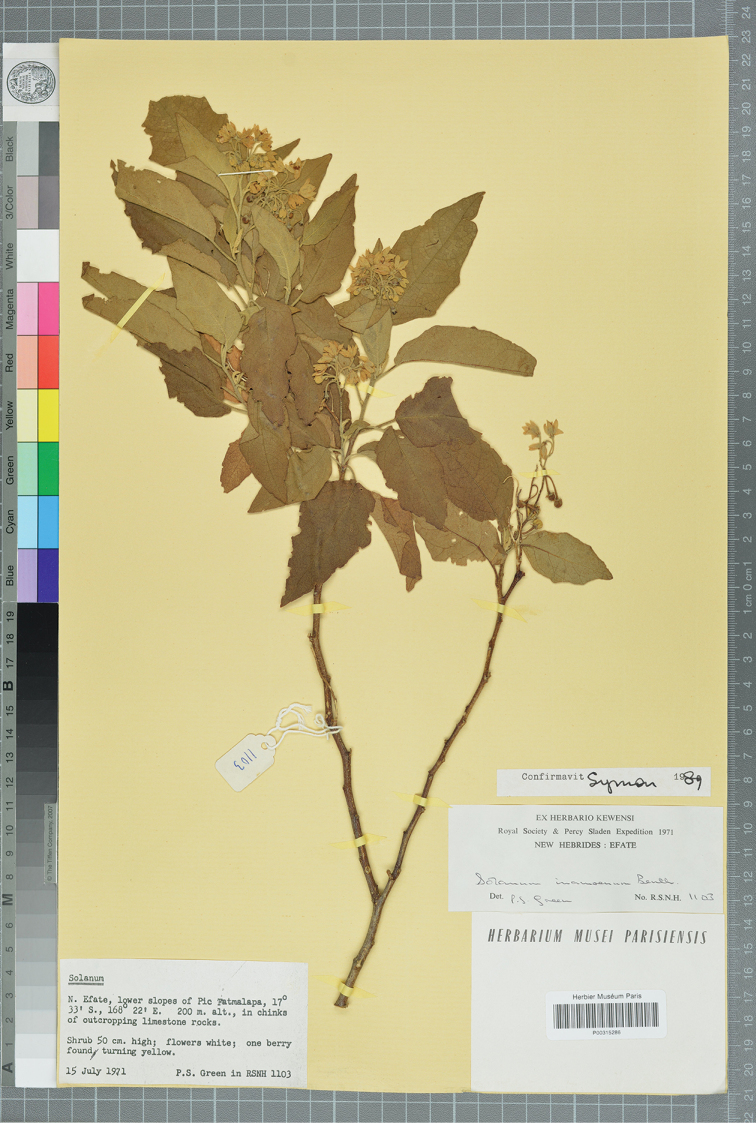
*Solanum vanuatuense* D.McClelland (holotype *Green 1103*, P [P00315286]). Reproduced with permission.

##### Type.

Vanuatu. Efate: lower slopes of Pic Fatamalapa, 17°33'S, 168°22'E, 200 m, 15 Jul 1971 (fl, fr), *P.S. Green 1103* (holotype: P [P00315286]; isotypes: A, K [K001155324], L [L0531790], NSW [acc. # 594210]).

##### Description.

Shrub to 2 m, the internodes to 5.1 cm long, unarmed. Stems densely pubescent with yellow to yellow-ferruginous, sessile and stalked porrect-stellate trichomes, the stalks of various lengths to 0.1 mm long, the rays 6–8, 0.2–0.3 mm long, the midpoint more or less equal to the rays; new growth densely pubescent with sessile or very short-stalked porrect-stellate trichomes and minute glandular hairs to 0.05 mm long; bark of older stems reddish brown. Sympodial units difoliate, the leaves geminate or not, if geminate the minor leaves 1/2–2/3 as large as the major leaves and similar in shape. Leaves simple or shallowly lobed; blades (major leaves) 4.9–11.2 cm long, 1.9–3.9 cm wide, 2.6–3 times as long as wide, lanceolate to ovate, chartaceous, discolorous; adaxial surfaces sparsely to moderately pubescent with short-stalked porrect-stellate trichomes, the stalks to 0.06 mm long, the rays 4–8, 0.15–0.25 mm long, the midpoint equal to or longer than the rays; abaxial surfaces sparsely to moderately pubescent with sessile or short-stalked porrect-stellate trichomes, the stalks to 0.3 mm long, the rays 4–8, 0.2–0.35 mm long, the midpoint equal to or longer than the rays; principal veins 4–6 pairs, the midrib raised abaxially and adaxially, the lateral veins weakly brochidodromous or semicraspedodromous, or craspedodromous in lobed leaves, raised abaxially, distinct adaxially; base rounded, typically oblique though occasionally equilateral; margins entire, sinuate, or shallowly lobed, the sinuses less than 1/4 of the distance to the midrib; apex acute or acuminate; petiole 6.2–16.8 mm long, 0.6–1.3 mm in diameter, densely stellate-pubescent with trichomes like those of the leaves, channeled above. Inflorescence to 8.9 cm, appearing lateral, extra-axillary, emerging from the middle or upper 1/3 of the internode, branched 1–3 times, with 60+ flowers, densely pubescent with sessile porrect-stellate trichomes; peduncle 0.3–1.9 cm long, 0.6–1.8 mm in diameter, unarmed; pedicels 0.7–1.0 cm long, ca. 0.3 mm in diameter at the base, 0.6–0.9 mm in diameter below the calyx, straight, not bent below the calyx or bent to approximately 90°, swelling more or less evenly from the base to the base of the calyx, densely pubescent, articulated at the base; pedicel scars congested to spaced 5.7 mm apart, overlapping to spaced 0.4 mm apart in the distal portion of the inflorescence, rigid, in two rows. Buds ovate, the calyx densely stellate-pubescent, the corolla densely stellate-pubescent where exposed abaxially, strongly exserted from the calyx before anthesis. Flowers 5-merous, all perfect or apparently so. Calyx 2.2–2.8 mm long, appearing nearly truncate with caudate lobe tips, the tube 0.5–0.9 mm long, the sinuses opaque when dry, the lobes 1.2–2.0 mm long, caudate, moderately to densely pubescent abaxially, glabrous adaxially, splitting in the sinuses during fruit development and then the lobes deltate. Corolla 1.1–1.3 cm in diameter, stellate, white, the interpetalar tissue well-developed, glabrous, the lobes 3.9–4.5 mm long, 2.2–3.5 mm wide, deltate, spreading at anthesis, adaxially glabrous or with a few scattered sessile porrect-stellate trichomes, densely pubescent abaxially. Stamens equal; filament tube minute; free portion of the filaments ca. 0.8 mm long, glabrous; anthers 2.5–2.9 mm long, 0.9–1.1 mm wide, markedly tapering, straight, yellow, spreading, poricidal at the tips, the pores directed dorsally, extending around the edge of the apex. Ovary ca. 0.6 mm, globose, moderately pubescent at the apex with simple glandular trichomes; style 5.6–6.4 mm long, ca. 0.2 mm in diameter, exserted from the anther cone, deflexed and emerging from between two adjacent stamens, filiform, curved or hooked at the apex, glabrous or sparsely pubescent on the basal 1/3 with simple glandular hairs; stigma 0.3–0.6 mm in diameter, capitate, minutely papillate. Fruit a globose juicy berry, 0.6–0.8 cm in diameter, apparently orange or red when mature, glabrous, the pericarp thin, glossy; fruiting calyx lobes 1.7–2.4 mm long, 1.3–1.5 mm wide, sparsely to moderately stellate-pubescent, appressed to the berry surface; fruiting pedicels 11.1–16.2 mm long, ca. 0.4 mm in diameter at the base, 1.3–1.7 mm in diameter below the calyx, straight, not bent below the calyx, gradually increasing in diameter in the distal 1/2–2/3, sparsely to moderately stellate-pubescent. Seeds 10–20 per fruit, 1.5–1.8 mm long, 1.5–2.0 mm wide, flattened-orbicular and notched at the point of attachment to flattened-reniform, red-brown when dry, the surface evenly reticulate, the testal cells with straight walls, the seed margins incrassate. Chromosome number not known.

##### Distribution and ecology

**Figure 13.**
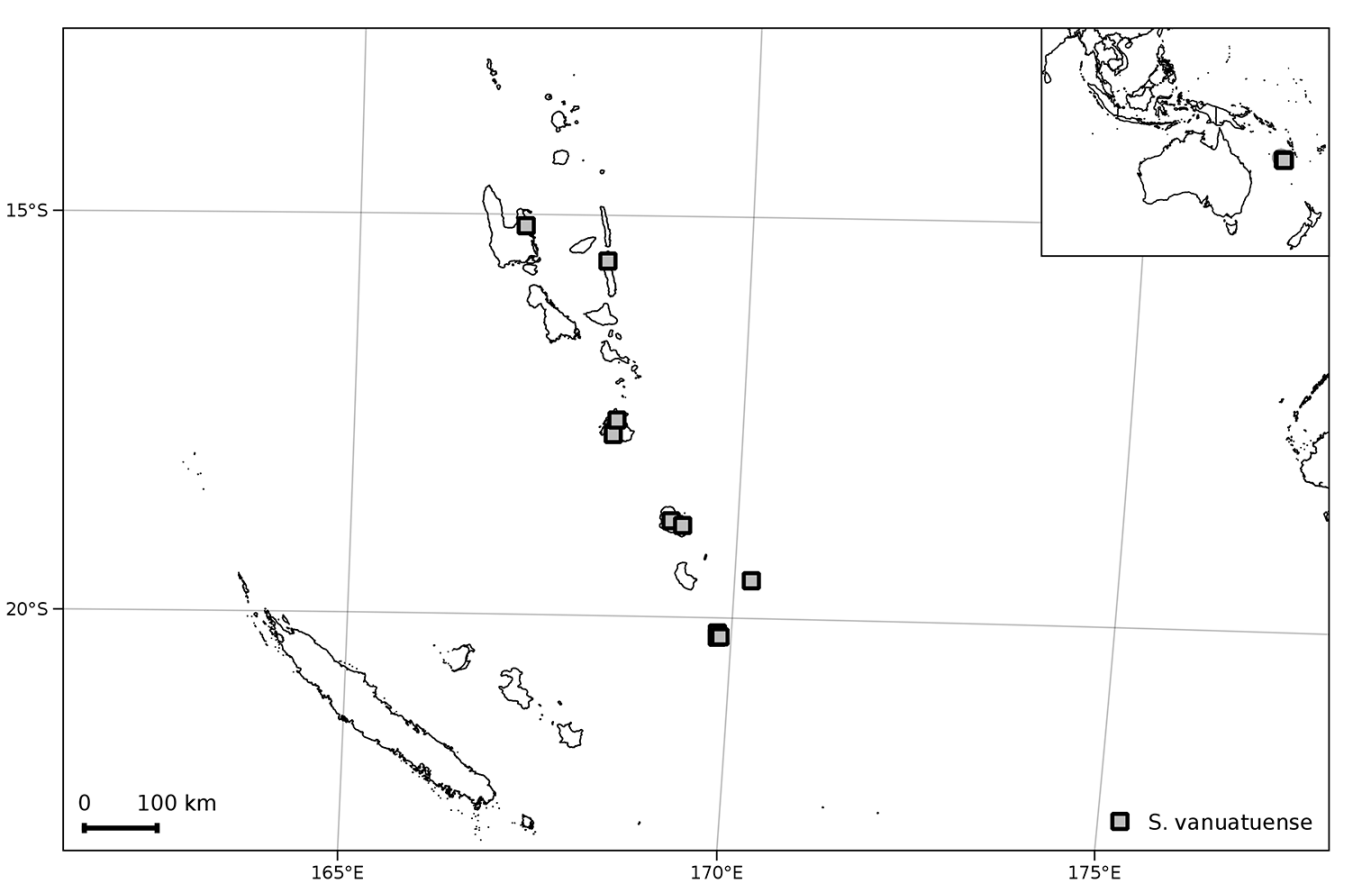
Distribution of *Solanum vanuatuense*.

(Figure 13). *Solanum vanuatuense* occurs on several islands in the Vanuatu archipelago; it is reported as growing in grass or low herbage, seaside, and on outcroppings of limestone from sea level to 200 m elevation.

##### Phenology.

Known to flower and fruit February, July, and October–November.

##### Etymology.

This species is named for island state of Vanuatu.

##### Preliminary conservation assessment

(IUCN 2019). EOO = 45,572 km^2^ [LC – Least Concern]; AOO = 40 km^2^ [EN –Endangered]. Although *Solanum vanuatuense* occurs on six islands in the archipelago, its coastal habitat is almost certainly threatened by human activities. We assign a preliminary status of EN (B2a,b) due to the fragmented nature of populations on different islands and threats to coastal habitats.

##### Discussion.

Specimens of *Solanum vanuatuense* were included in the sympatric *S. milnei* Seem. (Seemann 1866) in the original description of the latter; it differs from that species in its smaller flowers and geminate leaves that are often lobed. *Solanum vanuatuense* is also similar to *S. austrocaledonicum* Bitter of New Caledonia and Vanuatu from which it can be distinguished by its geminate leaves that are often deeply lobed, its deltate calyx lobes (rather than long apiculate on a truncate rim) and small flowers. *Solanum vanuatuense* and *S. austrocaledonicum* co-occur on the island of Anatom (*Schmid 5149* is *S. vanuatuense* and *Schmid 5149b* is *S. austrocaledonicum* – both specimens mounted on P00315283).

##### Additional specimens examined.

Vanuatu. Anatom: entre Umetch et Aneghowhat, 12 Feb 1986 (fl, fr), *Bourdy 442* (K, P); sin. loc., (fl) *McGillivray s.n.* (BM); secteur de Umec [Umetch], Nov 1974 (fl, fr), *Schmid 5149* (P). Efate: Port Vila, Oct 1883 (fl, fr), *Levat s.n.* (P). Erromango: entre Cook Bay et Ipota, ca. 5 m, 25 Jul 1983 (fl, fr), *Cabalion 2247* (K, P); tableland, 600 ft, 26 Jul 1930 (fl), *Cheeseman 38* (K). Espiritu Santo: Hog Harbour, 24 Nov 1933 (fl, fr), *Baker 58* (BM). Futuna: “tableland”, Dec 1858 (fl, fr), *Milne 391* (K). Pentecost Island: north end, ca. 1972, *Walsh 208* (NSW).

## Supplementary Material

XML Treatment for Solanum labyrinthinum

XML Treatment for Solanum peekelii

XML Treatment for Solanum caumii

XML Treatment for Solanum memaoyanum

XML Treatment for Solanum pseudopedunculatum

XML Treatment for Solanum ratale

XML Treatment for Solanum semisucculentum

XML Treatment for Solanum vanuatuense

## Supplementary materials

Supplementary material 1 File SM 1 Data type Specimen and locality data Explanation note CSV file of specimen and locality data (including georeferencing) for all taxa treated here. https://binary.pensoft.net/file/398922This dataset is made available under the Open Database License (http://opendatacommons.org/licenses/odbl/1.0/). The Open Database License (ODbL) is a license agreement intended to allow users to freely share, modify, and use this Dataset while maintaining this same freedom for others, provided that the original source and author(s) are credited.Donald H.R. McClelland, Michael Nee, Sandra Knapp
